# Elastin Insufficiency Confers Proximal and Distal Pulmonary Vasculopathy in Mice, Partially Remedied by the K_ATP_ Channel Opener Minoxidil: Considerations and Cautions for the Treatment of People With Williams-Beuren Syndrome

**DOI:** 10.3389/fcvm.2022.886813

**Published:** 2022-05-19

**Authors:** Russell H. Knutsen, Leah M. Gober, Elise K. Kronquist, Maninder Kaur, Danielle R. Donahue, Danielle Springer, Zu Xi Yu, Marcus Y. Chen, Yi-Ping Fu, Feri Choobdar, My-Le Nguyen, Sharon Osgood, Joy L. Freeman, Neelam Raja, Mark D. Levin, Beth A. Kozel

**Affiliations:** ^1^National Heart, Lung, and Blood Institute, National Institutes of Health, Bethesda, MD, United States; ^2^Mouse Imaging Facility, National Institute of Neurological Disorders and Stroke, National Institutes of Health, Bethesda, MD, United States; ^3^Murine Phenotyping Core, National Heart Lung and Blood Institute, National Institutes of Health, Bethesda, MD, United States

**Keywords:** pulmonary, elastin, minoxidil, Williams syndrome, vasculopathy, pulmonary artery, sex as a biological variable

## Abstract

**Background:**

Williams Beuren syndrome (WBS) is a recurrent microdeletion disorder that removes one copy of elastin (*ELN*), resulting in large artery vasculopathy. Early stenosis of the pulmonary vascular tree is common, but few data are available on longer-term implications of the condition.

**Methods:**

Computed tomography (CT) angiogram (*n* = 11) and echocardiogram (*n* = 20) were performed in children with WBS aged 3.4–17.8 years. Controls (*n* = 11, aged 4.4–16.8 years) also underwent echocardiogram. *Eln*^+/−^ mice were analyzed by invasive catheter, echocardiogram, micro-CT (μCT), histology, and pressure myography. We subsequently tested whether minoxidil resulted in improved pulmonary vascular endpoints.

**Results:**

WBS participants with a history of main or branch pulmonary artery (PA) stenosis requiring intervention continued to exhibit increased right ventricular systolic pressure (RVSP, echocardiogram) relative to their peers without intervention (*p* < 0.01), with no clear difference in PA size. Untreated *Eln*^+/−^ mice also show elevated RVSP by invasive catheterization (*p* < 0.0001), increased normalized right heart mass (*p* < 0.01) and reduced caliber branch PAs by pressure myography (*p* < 0.0001). *Eln*^+/−^ main PA medias are thickened histologically relative to *Eln*^+/+^ (*p* < 0.0001). Most *Eln*^+/−^ phenotypes are shared by both sexes, but PA medial thickness is substantially greater in *Eln*^+/−^ males (*p* < 0.001). *Eln*^+/−^ mice showed more acute proximal branching angles (*p* < 0.0001) and longer vascular segment lengths (*p* < 0.0001) (μCT), with genotype differences emerging by P7. Diminished PA acceleration time (*p* < 0.001) and systolic notching (*p* < 0.0001) were also observed in *Eln*^+/−^ echocardiography. Vascular casting plus μCT revealed longer generation-specific PA arcade length (*p* < 0.0001), with increased PA branching detectable by P90 (*p* < 0.0001). Post-weaning minoxidil decreased RVSP (*p* < 0.01) and normalized PA caliber (*p* < 0.0001) but not early-onset proximal branching angle or segment length, nor later-developing peripheral branch number.

**Conclusions:**

Vascular deficiencies beyond arterial caliber persist in individuals with WBS who have undergone PA stenosis intervention. Evaluation of *Eln*^+/−^ mice reveals complex vascular changes that affect the proximal and distal vasculatures. Minoxidil, given post-weaning, decreases RVSP and improves lumen diameter, but does not alter other earlier-onset vascular patterns. Our data suggest additional therapies including minoxidil could be a useful adjunct to surgical therapy, and future trials should be considered.

## Introduction

Elastin insufficiency produces distinctive vascular abnormalities that include both focal stenosis in proximal elastic arteries and general large vessel arteriopathy consisting of narrow, stiff blood vessels throughout the body. People with elastin insufficiency, such as those with Williams Beuren syndrome [WBS; as reviewed by Kozel, Barak (1)] have prominent vascular features. The pathognomonic lesion for diseases of elastin insufficiency is a focal stenosis of the ascending aorta, also known as supravalvar aortic stenosis (SVAS), but long segment stenosis also occurs commonly. The proximal pulmonary arteries, descending aorta and its branches may also be impacted by stenosis (2). Estimates vary by study methodology, but show pulmonary vascular abnormalities in ~40–60% of individuals with WBS (3, 4). They usually self-resolve in those with mild stenosis and some with “moderate” stenosis, but it's unclear whether those with resolving moderate stenosis were truly moderate (5, 6).

Less than half of individuals with pulmonary vascular abnormalities are thought to require surgical intervention. Catheter-based intervention has been successful in some cases, but surgical options are considered superior (7). Less is known about the impact and extent of peripheral pulmonary vascular lesions in WBS, as these vessels are more difficult to image with echocardiography and sometimes even with computed tomography (CT) angiography. Previous investigations in rodents showed potential for minoxidil, a K_ATP_ channel opener and vasodilator, to induce elastin production and generate remodeling that improved blood flow to organs in systemic circulation (8, 9). While a clinical trial with this medication did not show improvement in intima media thickness of the carotid artery, the primary endpoint for this study, an increase in lumen diameter was noted (10). Because pulmonary vascular abnormalities are particularly complex to treat through surgical intervention, we questioned whether minoxidil may add benefit to those receiving intervention for hemodynamically significant pulmonary vascular abnormalities.

To evaluate the impact of pulmonary vascular disease in elastin insufficiency, we examined elastin heterozygous (*Eln*^+/−^) mice. In addition to expanding previously published findings in the proximal vasculature using *in vivo* and *ex vivo* methods, we also investigated the impact of elastin deficiency in more distal vasculature over a broad developmental window. In addition, we focused on sex as a biological variable, given the propensity for vascular disease in WBS to be more severe in males (11). Finally, we tested the impact minoxidil has on the proximal and distal pulmonary vasculature and discussed the possibilities and limitations for using minoxidil to treat pulmonary vascular disease in WBS patients.

## Materials and Methods

### Human Subjects' Approvals and Inclusion

Oversight for the human subjects' research was provided by the Institutional Review Board of the National Institutes of Health. Data for all subjects and controls were obtained under the protocol ‘Impact of Elastin Mediated Vascular Stiffness on End Organs' (ClinicalTrials.gov Identifier: NCT02840448). Legal guardians for the pediatric patients signed consent. Assent was obtained from participants as appropriate. This study recruits people with WBS, WBS-like conditions and controls. Here, we analyze data from controls and those with WBS aged 3.4–17.8 years. To be considered for this analysis, an individual with WBS must have clinical or research molecular testing that confirms deletion of one copy of the *ELN* gene, using one of several methods equivalent to the clinically used fluorescent *in situ* hybridization (FISH) test for WBS. Not all participants were able to complete all tests.

### Human Echocardiographic Examination

Complete transthoracic echocardiograms (*n* = 48, 35 WS + 13 control) were performed using commercially available systems and measured according to American Society of Echocardiography guidelines. Portions of that data are reported elsewhere (12, 13). When available, tricuspid regurgitant (TR) peak velocity (TRV_max_) was recorded. It can be transformed using the modified Bernoulli equation to estimate RV systolic pressure RVSPe=4^*^(TRV_max_)^2^ + right atrial (RA) pressure. However, for simplicity, we report only the TRV_max_. If the initial research study failed to show a TR jet (*n* = 20, 18 WBS + 2 control), data from a second later research echocardiogram (*n* = 2 WBS) or concomitant clinical study (n=1 WBS) in which a jet was present were used instead, leading to 20 WBS and 11 control measures. In the past it has been assumed that most patients with clinically significant pulmonary hypertension would have a have measurable TRV jet (14, 15). As such, it may be safe to assume that RVSPe would not be significantly elevated in those participants (*N* = 1 WBS with history of PA intervention, *N* = 14 WBS with no history of PA intervention and *N* = 2 controls with no appreciable TR jet). Of note, the percentage of missing TRV_max_ was not different between the cases and controls and those with and without TRV_max_ measures were not statistically different in age, sex, and BMI distribution (data not shown).

### CT Analysis of Proximal and Peripheral Lung Vasculature

Pediatric participants with WBS underwent a neck to pelvis vascular contrast enhanced CT scan (Canon Aquilion ONE, Tokyo, Japan) with iopamidol-370 (Isovue-370, Bracco Diagnostics, Monroe Township, NJ). Images were reconstructed with 2 mm slice thickness and 1 mm slice increments.

Vitrea Advanced Visualization 7.11.5.29 (Vital Images Inc., Minnetonka, MN) was used to post-process and analyze compiled CT data. Vascular aorta mode was used to generate vessel cross sections with multi-planar reformatting. The midpoint of each vessel segment was identified using the ruler tool and electronic calipers were used to collect short and long axis lumen diameter measurements of the main, left, and right PAs.

Due to concerns about radiation exposure, pediatric controls were not scanned. Thus, PA diameters were converted to standardized effective diameter Z-scores and compared to a published control population (16). Briefly, predicted diameters based on participant height and sex were calculated and used to establish a Z-score. Not all children were able to lay still for the unsedated study.

### Animal Studies

All procedures described herein were approved by the Institutional Animal Care and Use Committee of the National Heart Lung and Blood Institute or Washington University School of Medicine. Institutional guidelines for animal experimentation and welfare were followed. The Eln^tm1Dyl^ mouse (17), was originally created in a 129x1/Sv; C57Bl/6 background but was further backcrossed to C57Bl/6 prior to these studies to remove 129x1/Sv genetic material that is known to influence phenotype (18). Studies were performed in male *Eln*^+/−^ and *Eln*^+/+^ mice ranging in age from postnatal (P) day ~1–90. Females were studied at ~P90. A subset of mice were treated with minoxidil (Sigma, St. Louis, MO) diluted in drinking water to provide ~20 mg/kg/day (8) from approximately postnatal day 21 to ~P90. Not all experiments could be performed in every mouse.

### Right Ventricular Hemodynamic Measurements

Mice were restrained on a heated holder to maintain body temperature, while anesthesia was induced at 2.5% isoflurane (Florane, Baxter, San Juan, PR). After induction anesthesia was reduced to 1.5%. Upon achieving a level plane of anesthesia, a pressure catheter (1.4-Fr, model SPR671, Millar Instruments, Houston, TX) was inserted into the jugular vein and advanced into the right ventricle (RV). At the time of pressure collection, anesthesia was further reduced to 1% and pressures were recorded using Chart 5 software (AD Instruments, Colorado Springs, CO). By closely observing the hemodynamic trace for a standard ventricular waveform, and monitoring physical resistance during catheter advancement, we were able to advance the catheter comfortably beyond the tricuspid valve and avoid over-advancing into the apex. This allowed for optimal catheter placement within the ventricular chamber. Animals were monitored for discomfort or over-sedation.

### Pressure-Diameter Testing

The left pulmonary artery (LPA), from the pulmonary trunk to just proximal to the first bifurcation, was excised post-euthanasia. Vessels were mounted on a pressure arteriograph (Danish Myotechnology, Copenhagen, Denmark) in balanced physiologic saline at 37°C, pressurized, and longitudinally stretched three times to *in vivo* length prior to data capture. Vessels were then transilluminated under a microscope connected to a charge-coupled device camera and computerized measurement system (Myoview, Danish Myotechnology) to allow for continuous recording of vessel diameters (further details on the pressure arteriography procedure can be found in (8). Intravascular pressure was increased from 0 to 70 mmHg in 10-mmHg steps. At each step, the outer diameter of the vessel was measured and manually recorded.

### Histology

Main pulmonary trunk arteries (MPA) were collected and fixed (unloaded) in 10% buffered formalin (Fisher Scientific, Waltham, MA) overnight at 4°C, and then progressively dehydrated in ethanol. Vessels were embedded in paraffin and 5μm cross-sections cut. Sections were subsequently stained using Verhoeff van Gieson stain (k059, Poly Scientific R&D, Bay Shore, NY) to visualize elastin. Slides were scanned using the Hamamatsu NanoZoomer 2.0-RS digital slide scanner. Images were captured at 40x with embedded scale using the Hamamatsu NDP.view2 viewing software (Hamamatsu Photonics, Hamamatsu, Japan). Lamellar number, circumference, and media wall thickness were measured as previously described (8).

### Cardiac Mass Measurements

Prior to sacrifice, body mass (BM) was recorded for each mouse. Post euthanasia, the heart was dissected from the chest cavity and the auricles removed. The heart was then carefully rolled on an absorbent surface to remove excess blood without crushing the tissue/disturbing the interstitial fluid. The RV was then detached from the left ventricle/septum (LV) and each segment was weighed separately. Using digital calipers, tibia length (TL) was recorded from the tibia head (exposed after the patellar tendon is severed) to the space within the ankle exposed after severing the Achilles tendon. The ratios of right ventricular heart mass (RVHM) and left ventricular/septal heart mass (LVHM) normalized to both body mass (RVHM/BM; LVHM/BM) and tibia length (RVHM/TL; LVHM/TL) were then calculated to assess relative changes in heart size.

### *In vivo* Murine Micro-Computed Tomography (CT) Imaging

Mice were placed in an induction chamber and anesthesia was induced using 4% isoflurane. Mice were transferred to a platform where a level plane of anesthesia at 2% isoflurane was achieved via nosecone. Ophthalmic ointment was applied and 80 microliters of Viscover™ ExiTron™ nano 12,000 contrast agent (Miltenyi Biotec, Bergisch-Gladbach, Germany) was delivered via standard tail vein injection. The platform with the secured mouse was transferred to the QuantumGX μCT chamber (PerkinElmer, Waltham, MA) and 4 min post-injection, the scan was initiated. A 4-min scan simultaneously capturing cardiac and respiratory rhythms was performed, and scans were reconstructed, gated to systole (19, 20).

### Murine *in vivo* μCT Image Analysis

To evaluate PA caliber, μCT scans (cardiac gated to systole) were uploaded to Horos (Horosproject.org, Annapolis, MD) and arterial caliber of the MPA, right pulmonary artery (RPA) and LPA were assessed using the 3D multiplanar reformation tool on an oblique slice. The pulmonary trunk was assessed distal to the valves, where the vessel becomes most uniformly circular. The RPA and LPA were assessed just distal to the branch point from the MPA. For all segments, the lumen diameter was measured in four evenly spaced intervals around the circumference of the vessel and averaged to account for variations in the vessel shape. The angle of branching between the RPA and LPA was assessed in an oblique slice where the trunk and both branching vessels were in view using the Horos angle tool. For all measures, still images were captured, and measurements were recorded.

### Pulmonary Artery and Left Ventricular Imaging by Echocardiogram

Cardiac imaging was performed using a high-frequency, high-resolution ultrasound system (Vevo2100, FUJIFILM VisualSonics Inc., Toronto, Canada) and a 40 MHz transducer probe (VisualSonics, MS-400). Mice were lightly anesthetized with isoflurane during the examination with HR maintained above 450 bpm. The mice were placed in a supine position over a heated imaging platform and rail system. Body temperature was maintained between 35 and 37 degrees Celsius as assessed by rectal probe. Color Doppler was applied to visualize PA flow from a modified long axis view; the parasternal long axis of the right ventricular outflow tract which visualizes the right ventricular outflow tract (RVOT), pulmonic valves and PA. Peak pulmonary artery velocity (mm/s), gradient (mm Hg), and pulmonary artery acceleration time (PAAT, s) were measured from the Pulsed Wave (PW) Doppler waveforms. PW Doppler samples were then taken parallel to MPA flow just distal to the pulmonic valve leaflets. Left ventricular measurements were made from the standard 2D and M mode images acquired from the parasternal long axis and mid-papillary short axis views.

### Polymer Infusion and μCT Imaging of Young and Adult Mice

Microfil^TM^ (Flow Tech Inc., Carver, MA) injections were performed as previously described (21). Approximate postnatal day 1 (P1), 7 (P7), 30 (P30), and 90 (P90) mice were used for these experiments. Briefly, after removal of the anterior chest wall and diaphragm, the PA was catheterized via the RV. The pulmonary vasculature was then flushed with phosphate buffered saline containing sodium nitroprusside (10^−4^ M) to clear and dilate the vessel network. The lungs were then inflated to 20 mm Hg following tracheal cannulation, while still in the body cavity. At this point, Microfil^TM^ was slowly infused into the arterial vascular network. Once the infusion was complete, the polymer was allowed to cure before the heart lung block was fixed in 10% formalin at 4°C for 24 h. The heart and lungs were then removed from the thoracic cavity *en bloc* and scanned by uCT. It should be noted that the inability to statically control intraluminal pressure during the delivery and polymerization of the Microfil^TM^ precludes accurate vessel diameter measures.

### *Ex vivo* μCT Imaging

Fixed mouse heart and lung samples were imaged using the Quantum GX μCT scanner (PerkinElmer, Waltham, MA). Based on field of view and specimen size, μCT scans were sub-reconstructed at 40μm (P90), 35μm (P30), 30μm (P7), or 25μm (P1). Both genotypes in each group were post-processed at the same resolution. Additional sub-reconstructions were used to achieve higher resolution for the left upper lobe with a focus on the left upper lobar feed artery (LULFA, see **Figures 7C,D**, arrowhead) at resolutions of 20μm (P90), 15μm (P30), 12μm (P7), and 10μm (P1), respectively.

### *Ex vivo* μCT – Proximal Vascular Analysis

To extract the proximal vasculature, sub-reconstructed images of heart/lung samples were loaded into Amira 6.7.0 (ThermoFisher Scientific). Segmentation was performed using ‘Magic Wand', a tool that connects all voxels within a manually denoted intensity range. A voxel at the center of the MPA was chosen as the “seed” and propagated to subsequent voxel connections of similar intensity. An upper threshold was then imposed on the reconstruction to simultaneously preserve the proximal conducting vasculature and exclude more distal vessels (see **Figures 7C,D**). Vessel length analysis was completed by selecting vessel segments stretching from the origin of the LPA or RPA and extending to the fourth branchpoint (Left Pulmonary Lobar Artery; LPLA) in the left lung and the sixth branchpoint (Right Pulmonary Lobar Artery; RPLA) in the right (see **Figures 7C,D** for details). “Graph Info” generated and compiled data for each vessel segment to measure path and Euclidean length along the extracted centerline. The ratio of path length to Euclidean length is defined as tortuosity. The branch angle formed between the LPA and RPA immediately as they branch off the MPA was also measured.

### *Ex vivo* μCT – Distal Vascular Tree Analysis

The higher resolution sub-reconstructions were used to examine the smaller vasculature of the LULFA, chosen for its consistent size, branching pattern, and even filling across samples. A seed voxel was planted in the main proximal artery feeding the LULFA. After propagation of the vasculature using ‘Magic Wand' and assignment of the volume to a material, the label image was created and skeletonized to find the centerline tree. To fully preserve the data from the smallest branches, the original vascular tree was not manually altered. Generation was assigned by selecting the LULFA's primary branch off the main artery and labeling it as the “root” of the tree, which allowed Amira to automatically assign increasing generations to the remainder of the tree. At each generation, the total length of vascular segments and total number of branches could then be quantified and compiled using “graph info.” The subsequent output yielded a single cumulative vascular arcade length and branch number for each generation. Data was an average of several mice/genotype at each generation.

### Statistics

Statistical analyses were performed using GraphPad Prism version 9 (GraphPad Software, San Diego, CA, www.graphpad.com.) One- or two-way ANOVA were employed as appropriate depending on the number of independent variables. The null hypothesis was rejected if ANOVA p<0.05 and multiple comparison testing was then performed with corrections for multiple testing by Tukey or Dunn (reported in figure legend for each graph). Comparison data are shown for pairwise testing based on genotype, sex, age or minoxidil treatment (Tx). Data for not scientifically relevant comparisons (e.g., *Eln*^+/+^ male vs. *Eln*^+/−^ female) were computed but not displayed.

For single variate analyses, unpaired *t*-tests were used for normally distributed data and Mann-Whitney testing was used with non-normally distributed data. Untreated *Eln*^+/−^ males in the sex-based experiments were also utilized as the untreated cohort in the minoxidil experiments.

Chi-square analysis was used to evaluate the relationship between genotype and the presence of a mid-systolic notch.

## Results

### Elevated RVSPe Persists in WBS Subjects Requiring Intervention

**Figure 1:**
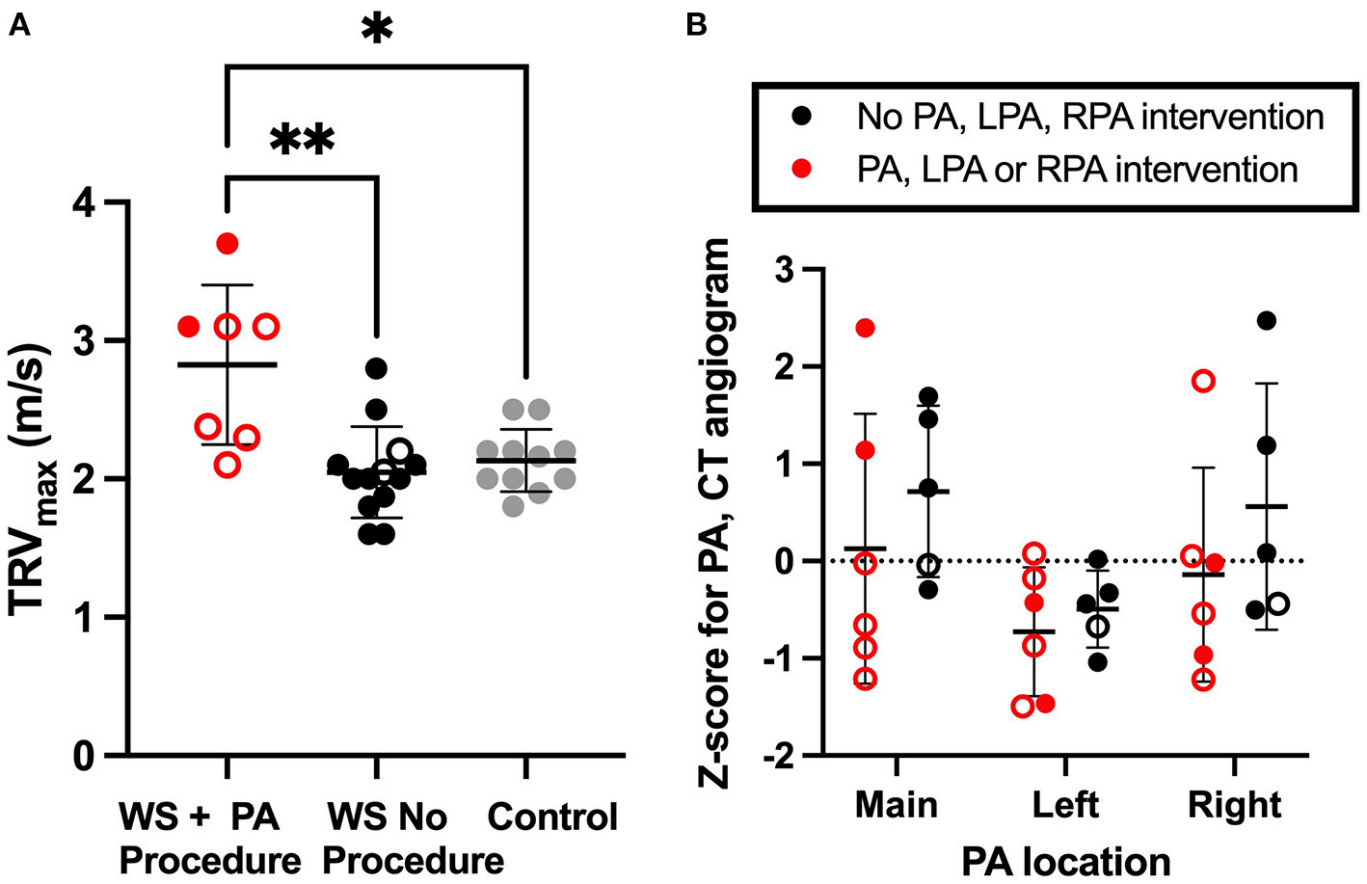
Residual increased right ventricular pressure estimate in WBS participants with history of pulmonary artery intervention. **(A)** shows the TRV_max_ recorded by echocardiogram. RVSPe can be approximated from TRV_max_ using Bernoulli's equation as RVSPe=4*(TRV_max_)^2^ + right atrial (RA) pressure. **(B)** illustrates Z-scored measures of main (MPA), left (LPA) and right (RPA) pulmonary artery lumen diameter. In both figures, WBS participants with a history of PA intervention are red, WBS participants with no PA intervention are black and controls are gray. A mean line +/- SD line is shown for each group. Open circles represent individuals who had SVAS repair. Closed circles are used for those not requiring SVAS repair. Multiple comparison testing (after ANOVA) by Tukey. * *p* < 0.05, ** *p* < 0.01.

To further evaluate pulmonary vascular status in WBS, we performed echocardiograms and CT angiograms with IV contrast in subjects electing to undergo these procedures. See Supplementary Table 1 for demographic information. Briefly, complete echocardiogram with doppler was performed on 35 children with WBS and 13 controls. Of those, 20 echocardiograms from WBS participants and 11 echocardiograms from controls exhibited a measurable TRV jet to approximate RVSPe (see methods) for analysis in Fig. 1A. The resulting groups were of similar age, sex at birth, and BMI. Thirty-five percent of those with WBS (7 children) had previously undergone a pulmonary intervention.

TRV_max_ was elevated among children with WBS who had undergone PA reconstruction, compared to those with WBS who hadn't had the procedure and healthy controls (Figure 1A, Kruskal-Wallis ANOVA *p* < 0.01, followed by multiple comparison testing by Dunn's, WBS with history of PA procedure median (interquartile range, IQR) = 3.1 (0.8) m/s; WBS without history of PA procedure = 2.0 (0.3) m/s; Control = 2.2 (0.2) m/s). In this respect, we saw a bi-modal distribution, with four WBS subjects with history of PA procedure having very significantly elevated TRV_max_, including one subject who had undergone extensive PA reconstruction beyond the hilum and was planned for follow up surgical procedure. Following this study, that patient underwent clinically indicated cardiac catherization with balloon dilation angioplasty, which did result in significantly diminished MPA pressures (29/13/20 mmHg) with deliberate fracture of previously placed stents in the RPA and LPA, later reflected on echocardiogram by diminished TRV_max_ to 2.6 m/s. That subject's exertional fatigue symptoms resolved following intervention and planned follow up surgeries were delayed for 1–2 years. TRV_max_ measures for subjects who did not undergo PA reconstruction was not significantly different from matched control (Dunn).

Right and left branch pulmonary artery diameter was quantified from cardiac gated CT angiograms and converted to Z-score (9), and groups compared by two-way ANOVA (Figure 1B). No statistically significant pulmonary artery caliber dimension Z-score difference was seen between those who did or did not have PA reconstruction surgery.

### Elastin Insufficiency Induces Increased RV Systolic Pressure in Male and Female Mice and Increased Right Heart Mass to Tibial Length That Is More Severe in Females

**Figure 2:**
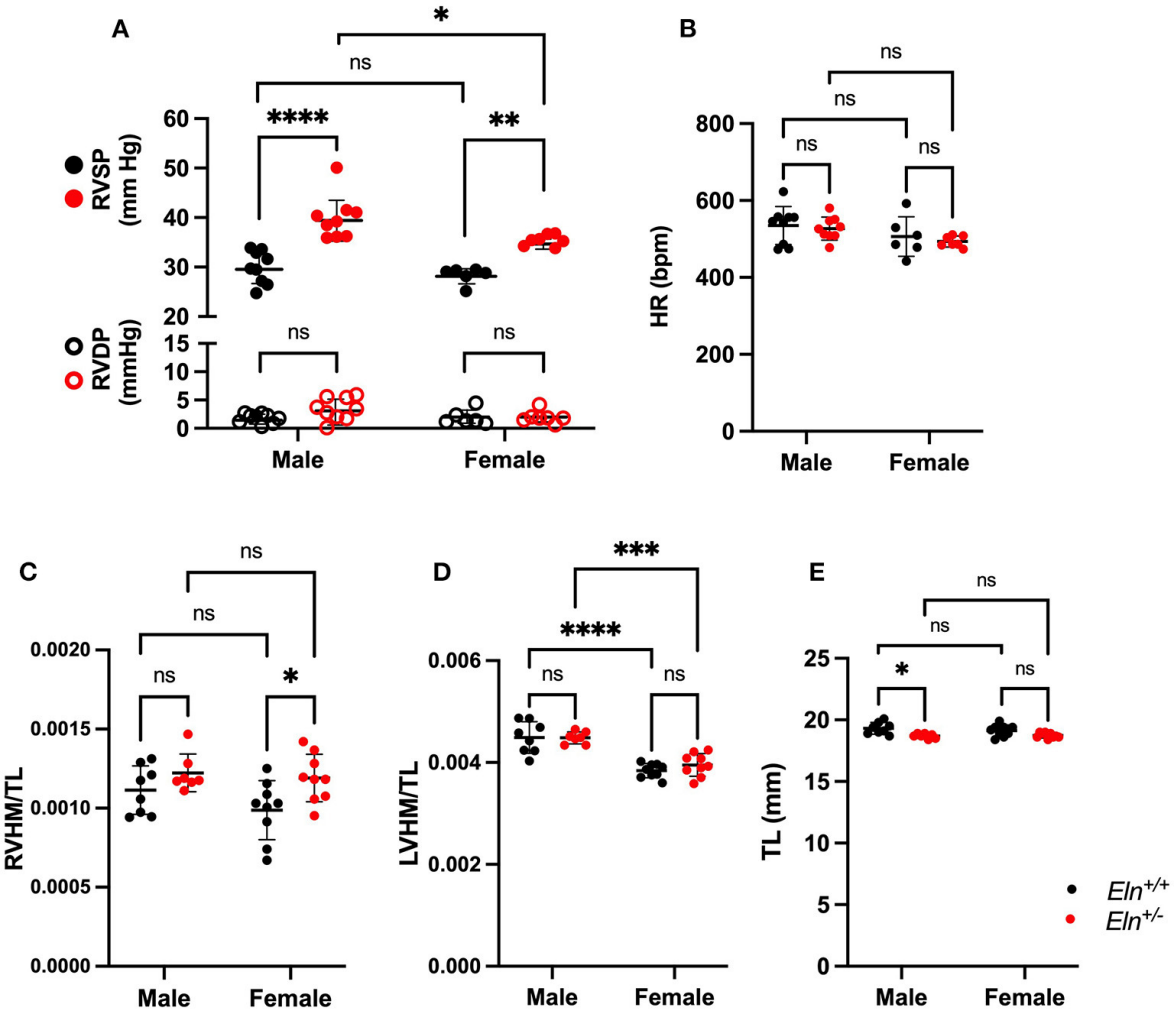
Sex and Eln genotype differences in hemodynamic parameters. **(A)** shows RVSP in closed circles and RVDP in open circles in male and female *Eln*^+/+^ and *Eln*^+/−^ mice. **(B)** depicts HR in the same groups. **(C–E)** report heart mass as normalized to tibial length findings. **(C)**: RVHM/TL, **(D)**: LVHM/TL (this measure includes the interventricular septum and the LV in the LVHM measure), **(E)**: tibial length. *Eln*^+/+^ data are presented in black and *Eln*^+/−^ in red in all figures. A mean line +/- SD is shown for each sex/genotype combination. Multiple comparison testing (after ANOVA) by Tukey. * *p* < 0.05, ** *p* < 0.01, *** *p* < 0.001, **** *p* < 0.0001, and ns is not significant.

Pulmonary pressures were first assessed by invasive catheter measurement in untreated 3-month-old male and female mice. Right ventricular systolic pressure (RVSP) was higher in *Eln*^+/−^; (two-way ANOVA, genotype effect, *p* < 0.0001; Figure 2A) and in males (sex effect: *p* < 0.05), with no sex X genotype interactive effect. Multiple comparison testing showed RVSP was significantly elevated in both male (9.9 mmHg higher, *p* < 0.0001) and female (7.1 mmHg higher, *p* < 0.01) *Eln*^+/−^ mice as compared to same sex *Eln*^+/+^ mice (Figure 2A). Additionally, RVSP was significantly elevated in male *Eln*^+/−^ compared to female *Eln*^+/−^ mice (4.5 mmHg higher, *p* < 0.05). There were no statistically significant differences between *Eln*^+/+^ males and females. There were not statistically significant differences in right ventricular end-diastolic pressure (RVDP) by two-way ANOVA (Figure 2A).

Heart rate (HR) was higher in males (two-way ANOVA, sex effect, *p* < 0.05; Figure 2B) but was not different based on Eln genotype; no interactive effects were identified. Multiple comparison testing revealed no statistically significant differences between genotype or sex-based pairs.

The right ventricular heart mass to tibia length (RVHM/TL) ratio was higher in *Eln*^+/−^ mice, (two-way ANOVA, genotype effect, *p* < 0.01; Figure 2C), but there were no sex or sex X genotype interactive effects. On multiple comparisons testing, female *Eln*^+/−^ mice exhibit elevated RVHM/TL ratios compared to female *Eln*^+/+^ (*p* < 0.05; Figure 2C), but the male comparisons were not significantly different and there were no differences in male-female pairs of the same *Eln* genotype. The left heart, by comparison, showed higher heart mass to tibia length ratio (LVHM/TL) in males (two-way ANOVA, sex effect, *p* 0.0001; Figure 2D), but no genotype or sex X genotype interactive effect. In this case, both *Eln*^+/+^ and *Eln*^+/−^ males had larger LVHM/TL ratios than genotype matched females (*p* < 0.0001 for *Eln*^+/+^ and *p* < 0.001 in *Eln*^+/−^). Some of the cardiac differences may have been influenced by genotype-mediated differences in tibia length (*Eln*^+/−^ had shorter TL *p* < 0.001; Figure 2E; no sex or sex X genotype interactive effects for TL) in the normalization. Multiple-comparisons testing show male *Eln*^+/−^ mice have significantly smaller tibias than male *Eln*^+/+^ mice (*p* < 0.05), with the female comparison trending smaller, but not significant. When heart mass is instead normalized to body mass, RVHM/BM is again higher in *Eln*^+/−^ mice (two-way ANOVA, genotype effect, *p* < 0.01; Supplementary Figure 1A), but no sex or sex X genotype interactive effects are seen. On multiple comparisons testing, female *Eln*^+/−^ mice exhibit elevated RVHM/BM ratios compared to female *Eln*^+/+^ (*p* < 0.05; Supplementary Figure 1A), but the male comparisons were not significantly different and there were no differences in male-female pairs of the same *Eln* genotype. LVHM/BM, in contrast, showed no effects by two-way ANOVA (Supplementary Figure 1B). Predictably, female mice weighed less than males (two-way ANOVA, *p* < 0.0001; Supplementary Figure 1C). There were no genotype or interactive effects. Multiple comparisons testing confirms male *Eln*^+/+^ (*p* < 0.001) and *Eln*^+/−^ (*p* < 0.001; Supplementary Figure 1C) are significantly larger than females of the same genotype.

### Elastin Insufficiency Produces Decreased PA Diameter With Increased Wall Thickness That Is Most Severe in Male *Eln^+/−^* Mice

**Figure 3:**
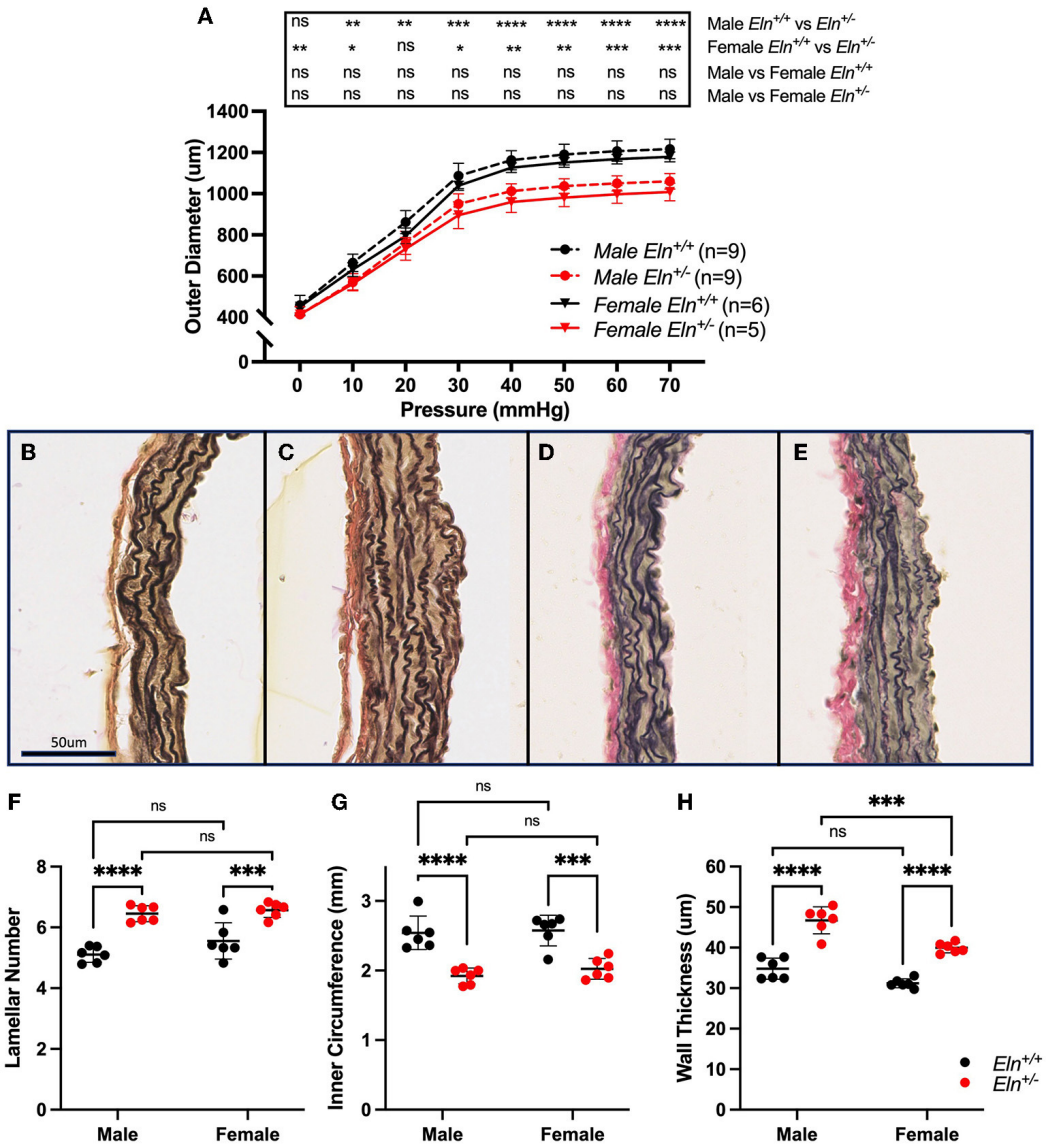
Sex and Eln genotype differences in pulmonary artery morphology. **(A)** reports the relationship between intravascular pressure (x axis) and LPA diameter (y axis). **(B–E)** show VVG stained images of male *Eln*^+/+^ **(B)**, male *Eln*^+/−^ **(C)**, female Eln^+/+^ **(D)**, and female *Eln*^+/−^ **(E)** main pulmonary artery. **(F)** shows lamellar number. **(G)** is the MPA internal medial circumference and **(H)** shows the medial thickness for the various sex-genotype groups. *Eln*^+/+^ data are presented in black and *Eln*^+/−^ in red in all figures. In **(A)**, mean +/- SD are shown for all points. Males are shown as dashed lines and females as solid lines. For **(F–H)**, a mean line +/- SD is shown for each sex/genotype combination. Multiple comparison testing (after ANOVA) by Tukey. * *p* < 0.05, ** *p* < 0.01, *** *p* < 0.001, **** *p* < 0.0001, and ns is not significant.

To assess PA diameter, LPAs were excised and evaluated on a pressure myograph. In male and female cohorts, multiple-comparisons testing following two-way ANOVA (group effect, pressure effect and pressure X group interactive effect all *p* < 0.0001) showed consistently decreased *Eln*^+/−^ outer diameters compared to *Eln*^+/+^ (Figure 3A). There were no significant differences between sexes of the same genotype.

Histologic sections from unloaded male *Eln*^+/+^ (Figure 3B), male *Eln*^+/−^ (Figure 3C), female *Eln*^+/+^ (Figure 3D), and female *Eln*^+/−^ (Figure 3E) MPAs were assessed for structural differences. More elastic lamellae are seen in *Eln*^+/−^ (two-way ANOVA, genotype effect, *p* < 0.0001; Figure 3F), but no sex or sex X genotype interactive effects were seen. Multiple-comparison testing confirmed male (*p* < 0.0001) and female (*p* < 0.001) *Eln*^+/−^ have significantly more elastic lamellae than same sex *Eln*^+/+^, but there were no statistically significant differences between genotype-based pairs.

MPA internal medial circumference, a proxy for unloaded lumen size, was smaller in *Eln*^+/−^ mice (two-way ANOVA, genotype effect, *p* < 0.0001; Figure 3G), without sex or interactive effect. Multiple-comparison testing confirmed *Eln*^+/−^ inner circumference is smaller than *Eln*^+/+^ in both males (*p* < 0.0001) and females (*p* < 0.001, Figure 3G), but there were no statistically significant differences between genotype-based pairs.

Interestingly, media thickness was increased in *Eln*^+/−^ as compared to *Eln*^+/+^ (two-way ANOVA, genotype effect, *p* < 0.0001) and in males compared to females (sex effect *p* < 0.001) effects; no interactive effects were seen (Figure 3H). MPA wall thickness was increased in male *Eln*^+/−^ (*p* < 0.0001, Fig 3H) and female *Eln*^+/−^ (*p* < 0.0001) compared to their sex matched *Eln*^+/+^ counterparts. However, male *Eln*^+/−^ MPA medial wall thickness was also notably thicker than female *Eln*^+/−^ (*p* < 0.001). A trend toward increased media thickness in male vs. female controls was noted as well (*p* = 0.06, males thicker).

### Treatment With Minoxidil Increases *ex vivo* and *in vivo* Pulmonary Artery Caliber in *Eln^+/−^* Mice

**Figure 4:**
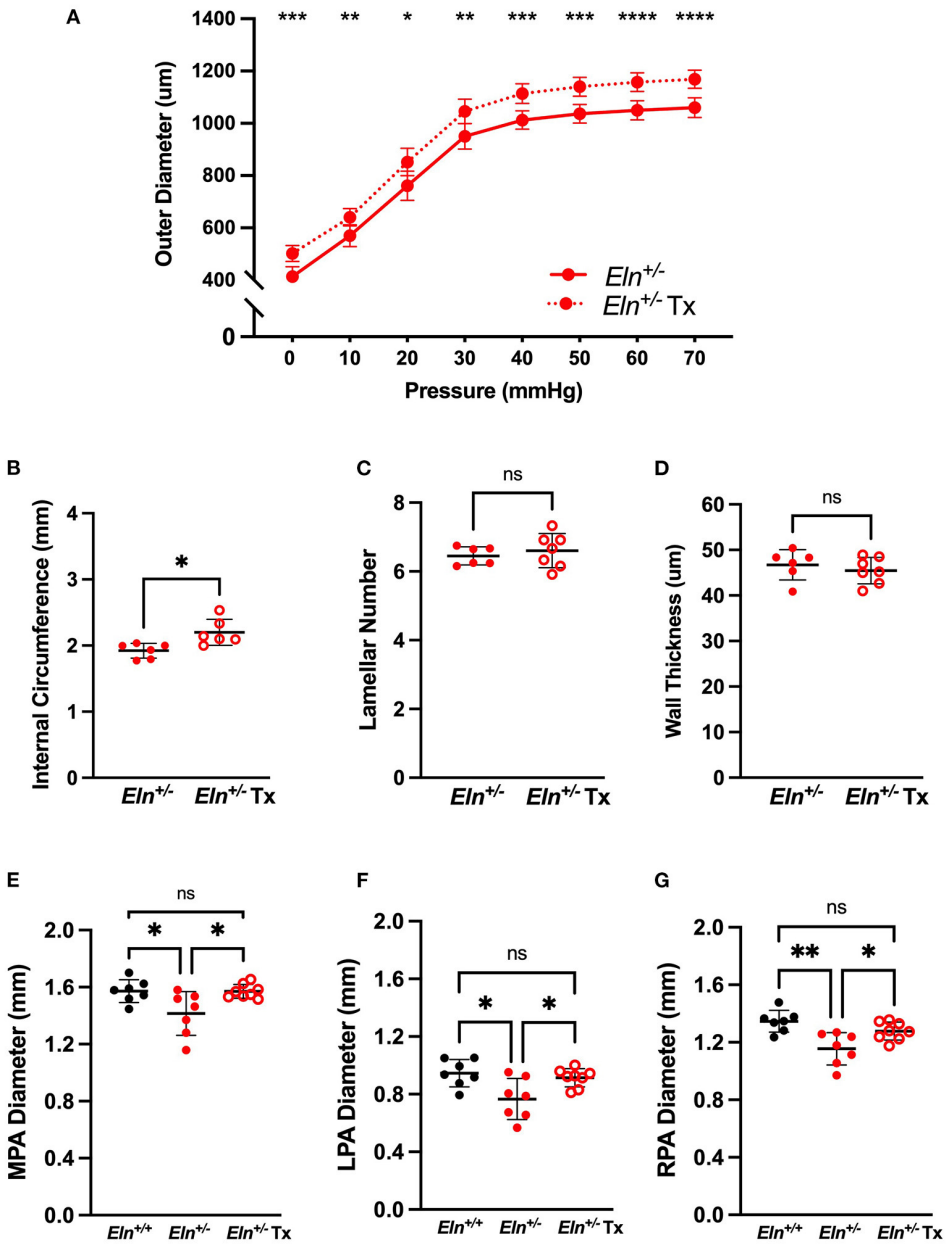
Minoxidil increases pulmonary artery caliber. **(A)** reports the relationship between intravascular pressure (x axis) and LPA diameter (y axis) for treated and untreated *Eln*^+/−^ mice. **(B–D)** depict measures collected from unloaded histological sections of main pulmonary arteries. **(B)** shows the internal medial circumference. **(C)** is lamellar number and **(D)** shows the medial thickness. **(E–G)** show *in vivo* arterial diameter measures for the main **(E)**, left **(F)** and right **(G)** pulmonary arteries. *Eln*^+/+^ data are presented in black and *Eln*^+/−^ in red. Untreated mice are depicted with closed circles and treated (Tx) mice are shown with open circles. In **(A)**, mean +/- SD are shown for all points. A mean line +/- SD is shown for each treatment/genotype combination in **(B–G)**. Unpaired *t*-tests were used in **(B–D)**. Multiple comparison testing (after ANOVA) by Tukey [**(A)** and **(E–G)**]. * *p* < 0.05, ** *p* < 0.01, *** *p* < 0.001, **** *p* < 0.0001 and ns is not significant.

To test minoxidil's therapeutic potential on the pulmonary vasculature, treatment (Tx) was initiated in *Eln*^+/−^ mice immediately post-weaning (~P21) to ~P90. Because no interactive effect was seen for sex for any of the measures tested, Tx was evaluated in male mice only. Consistent with aortic studies, minoxidil appeared to increase PA outer diameter at all pressures as measured by pressure myography (two-way repeated measures ANOVA, treatment effect, *p* < 0.0001; Figure 4A). Higher pressure was associated with increased diameter in all groups (pressure effect, *p* < 0.0001). There was also a borderline interaction (*p* 0.06) between Tx and pressure for PA outer diameter, with higher pressures generating greater differences between the treated and untreated cohorts. Multiple-comparisons testing showed that the treated *Eln*^+/−^ cohort had larger PAs compared to untreated *Eln*^+/−^ mice, across all pressures (Figure 4A).

While histology confirmed increased internal circumference in treated male *Eln*^+/−^ vessels compared to untreated *Eln*^+/−^ (unpaired *t*-test, *p* < 0.05; Figure 4B), Tx did not alter the established lamellar number (Figure 4C) or medial thickness (Figure 4D).

In parallel with the *ex vivo* myography studies, we also performed *in vivo* analyses using cardiac gated μCT angiogram. Following confirmation of a statistically significant genotype effect by one-way ANOVA, (MPA: *p* < 0.05, LPA: *p* < 0.01 and RPA: *p* < 0.01), multiple comparisons confirmed smaller lumen diameter in untreated *Eln*^+/−^ than *Eln*^+/+^ PAs, across all three vessels (MPA: *p* < 0.05, Figure 4E; LPA: *p* < 0.05, Figure 4F; RPA: *p* < 0.01, Figure 4G). Tx results in PA lumen diameter increase in treated *Eln*^+/−^ vessels compared to untreated *Eln*^+/−^ across all three vessels (MPA: *p* < 0.05; LPA *p* < 0.05; RPA *p* < 0.05). No pairwise differences were seen between *Eln*^+/+^ and treated *Eln*^+/−^ in any vessel type.

### Minoxidil Reduces RVSP as Measured by Invasive Pressure Catheterization in *Eln^+/−^* Mice

**Figure 5:**
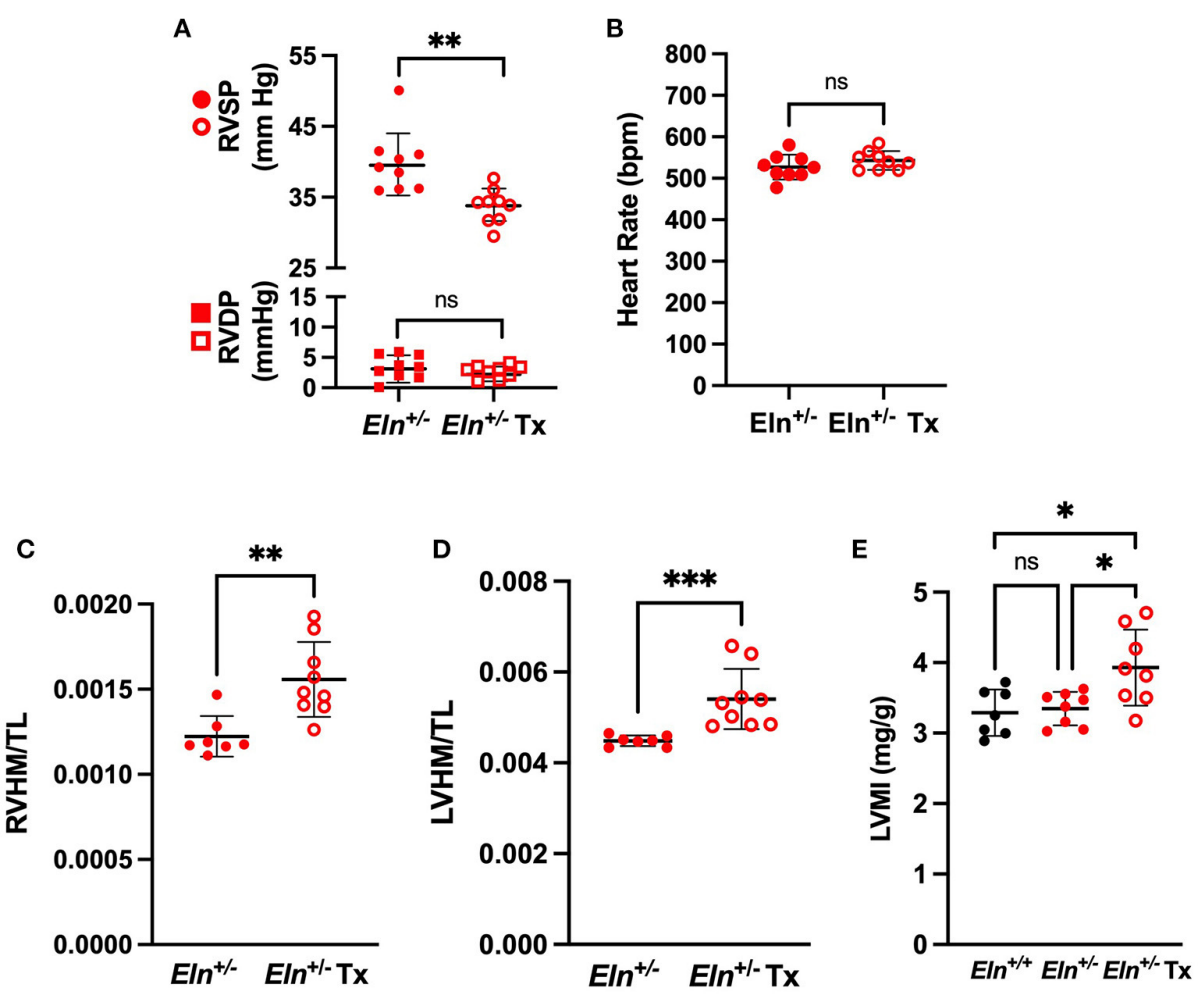
Minoxidil reduces right ventricular pressure and increases heart size in *Eln*^+/−^ mice. **(A)** shows RVSP in circles and RVDP in squares in treated and untreated *Eln*^+/−^ mice. **(B)** depicts HR in the same groups. **(C,D)** report heart mass as normalized to tibial length findings. **(C)**: RVHM/TL and **(D)**: LVHM/TL (this measure includes the interventricular septum and the LV in the LVHM measure). **(E)** depicts Estimated Left Ventricular Mass Index (LVMI) assessed using echocardiogram. *Eln*^+/+^ data are presented in black and *Eln*^+/−^ are in red. Untreated mice are depicted with closed circles and minoxidil treated (Tx) mice are shown with open circles. A mean line +/- SD is shown for each Tx/genotype combination. Unpaired *t*-test was used for **(A)** and **(B)**, Mann-Whitney for **(C)** and **(D)**, and one-way ANOVA for **(E)**. Multiple comparison testing (after ANOVA) by Tukey. * *p* < 0.05, ** *p* < 0.01, *** *p* < 0.001, and ns is not significant.

In the *Eln*^+/−^ cohort, Tx reduced RVSP (6.1 mmHg difference, unpaired *t*-test, *p* < 0.01; Figure 5A) as measured by invasive pressure catheterization and yielded no difference in RVDP (Figure 5A). No Tx effect was detected in *Eln*^+/−^ HR (Figure 5B).

Despite an apparent reduction of ventricular pressure and lumen size, minoxidil increased both RVHM/TL (Mann-Whitney; *p* < 0.01; Figure 5C) and LVHM/TL (Mann-Whitney, *p* < 0.001; Figure 5D) in *Eln*^+/−^ mice. There was no difference in tibia length between male *Eln*^+/−^ and *Eln*^+/−^ Tx (Mann-Whitney; Supplementary Figure 2A). Similar effects were seen in RV with normalization to body mass: Tx increased both RVHM/BM (Mann-Whitney, *p* < 0.01; Supplementary Figure 2B) and LVHM/BM (Mann-Whitney, *p* < 0.01; Supplementary Figure 2C) in *Eln*^+/−^ mice with no apparent difference in body mass identified between groups (Mann-Whitney; Supplementary Figure 2D).

Echocardiogram demonstrated similar findings. Following confirmation of statistically significant group effects for the echocardiogram measures by one-way ANOVA (data not reported), we performed pairwise comparisons. Echocardiogram estimated left ventricular heart mass indexed to body mass (LVMI) was indeed larger in the *Eln*^+/−^ Tx group than in either the *Eln*^+/+^ or the untreated *Eln*^+/−^ groups (p<0.05 for both comparisons, Figure 5E). Individual measures of wall thickness (left ventricular posterior wall thickness at systole (LVPWs) and interventricular septal wall thickness at systole (IVSs)) were also detectably larger in the *Eln*^+/−^ Tx group (See Supplementary Table 2 for details). Left ventricular chamber end diastolic dimension (LVIDd) was also larger in the treated *Eln*^+/−^ Tx mice as compared to *Eln*^+/+^ (*p* < 0.05; Supplementary Table) but was not larger than untreated *Eln*^+/−^.

### *Eln^+/−^* Mice Exhibit Systolic Notching and Reduced Pulmonary Artery Acceleration Time (PAAT) by Echo Doppler; Neither Resolve With Minoxidil

**Figure 6:**
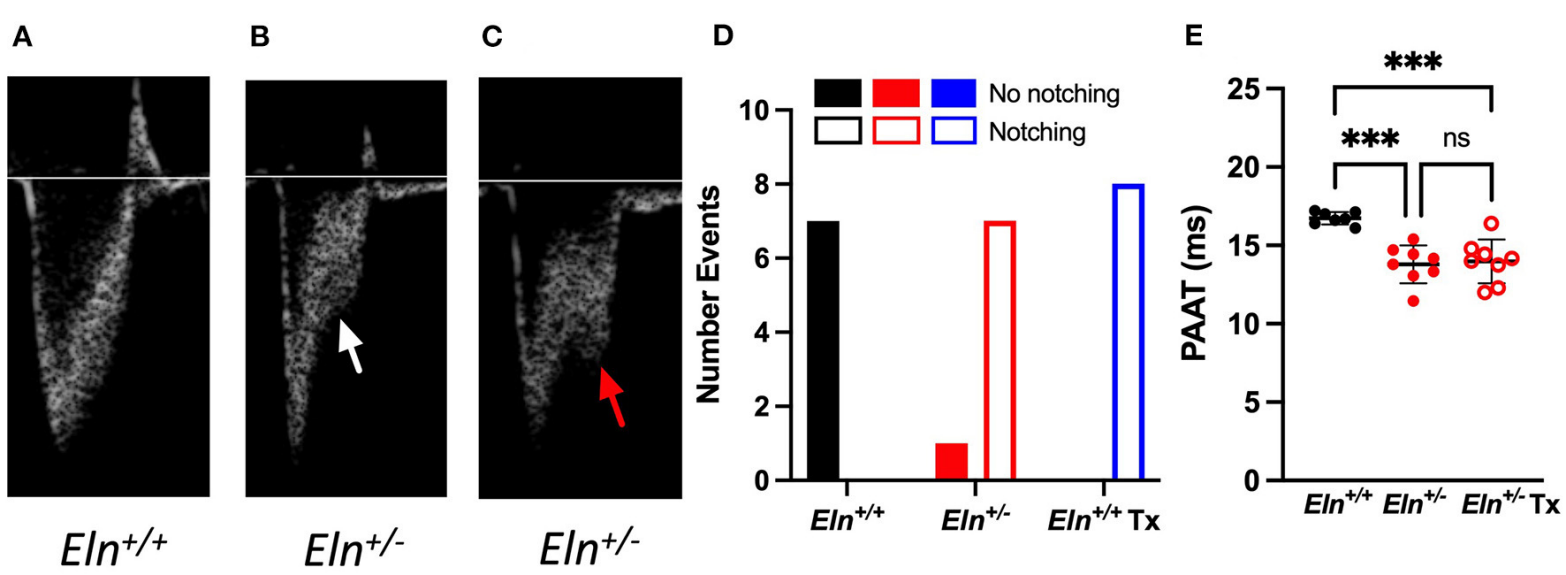
*Eln*^+/−^ mice display evidence of increased vascular resistance, not impacted by minoxidil. Panels A-C show pulmonary artery pulse wave Doppler. The typical parabolic trace is seen in the *Eln*^+/+^ **(A)**, while a shelf-like systolic notch is present in the majority of treated and untreated *Eln*^+/−^ mice **(B)** white arrow. In some cases, a second distinct systolic inflection, consistent with human systolic notching, becomes apparent **(C)** red arrow. Chi-square analysis **(D)** reports the frequency of notching in this cohort. **(E)** shows pulmonary artery acceleration time (PAAT). PAAT is associated with elevated pulmonary pressure and vascular resistance. In **(D)**, *Eln*^+/+^ data are presented in black, untreated *Eln*^+/−^ in red, and treated *Eln*^+/−^ in blue. Mice without a notch are shown in solid bars and those with systolic notching have open bars. In **(E)**, *Eln*^+/+^ data are presented in black and *Eln*^+/−^ are in red. Untreated mice are depicted with closed circles and minoxidil treated (Tx) mice are shown with open circles. A mean line +/- SD is shown for each Tx/genotype combination. Multiple comparison testing (after ANOVA) by Tukey. *** *p* < 0.001 and ns is not significant.

Notching is thought to be caused by an abnormal wave reflection that occurs in the setting of elevated PA impedance (22). Compared to the traditional parabolic trace in the *Eln*^+/+^ (Figure 6A), we see the emergence of systolic notching in the *Eln*^+/−^. This observation consistently presents as either a distinct “shelf” (white arrow, Figure 6B) or a second inflection (red arrow, Figure 6C). A notch was present in 0 out of 7 *Eln*^+/+^, 7 of 8 *Eln*^+/−^, and 8 of 8 *Eln*^+/−^ Tx echocardiograms (Chi-Square, *p* < 0.0001 χ^2^; Figure 6D), suggesting increased PA impedence. Diminished echocardiogram-based PAAT is an indication of elevated pulmonary systolic blood pressure and may imply increased resistance (23). Multiple comparison testing following one-way ANOVA (*p* < 0.001) shows that untreated *Eln*^+/−^ mice have decreased PAAT relative to *Eln*^+/+^ (*p* < 0.001; Figure 6E), but minoxidil does not rescue that decrease in the *Eln*^+/−^ Tx group

### Branching Patterns and Vessel Lengths of the Main Conducting Vessels Are Altered in the Eln+/-

**Figure 7:**
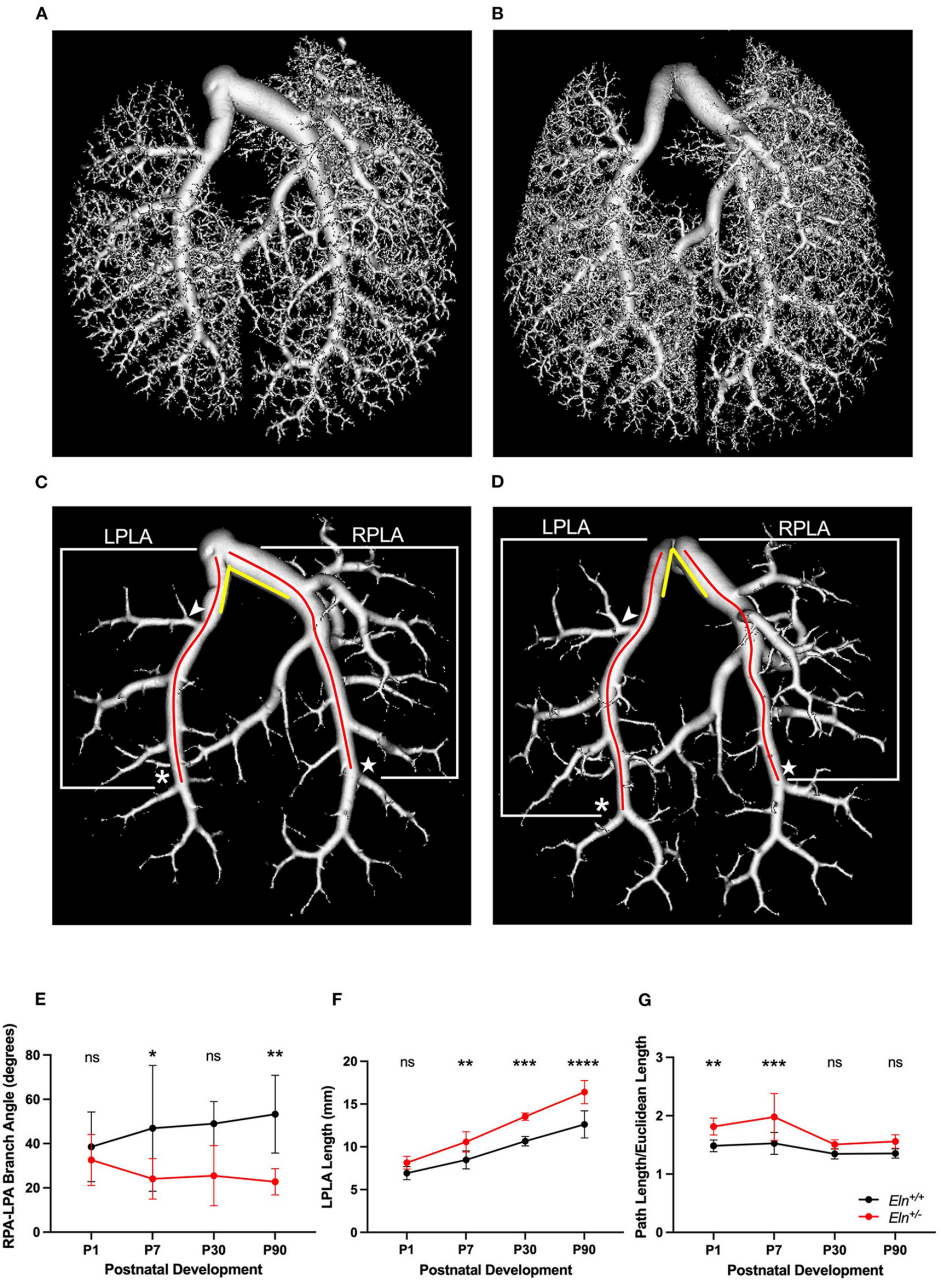
Elastin insufficiency reduces PA branching angle and increases proximal arterial segment length. **(A)** (*Eln*^+/+^) and **(B)** (*Eln*^+/−^) contain a volume reconstruction of a lung set generated through *ex vivo* vascular casting and microCT. **(C)** (*Eln*^+/+^) and **(D)** (*Eln*^+/−^) are the same lung sets thresholded for large vessel analysis. The LPLA (asterisk denotes fourth branch) and RPLA (star marks the sixth branch) are noted with red lines to illustrate a typical path length measured by the software for each vessel. An arrowhead notes the position of the LULFA for small vessel analysis (see Figure 8) and yellow lines are an approximation of the angle formed between the LPA and RPA. This branch angle is evaluated in **(E)** at P1, P7, P30, and P90. The LPLA length **(F)** and tortuosity **(G)** are shown at the same time points. In **(E–G)**, *Eln*^+/+^ data are presented in black and *Eln*^+/−^ are in red. A mean line +/- SD are shown for each age/genotype combination. Multiple comparison testing (after ANOVA) by Sidak. * *p* < 0.05, ** *p* < 0.01, *** *p* < 0.001, **** *p* < 0.0001 and ns is not significant (*n* = 5–8/group).

To evaluate phenotypic features that might influence vascular resistance, we utilized a polymer casting technique we recently optimized (21) to visualize the length and branching pattern of the pulmonary architecture using μCT at several developmental timepoints. A representative *Eln*^+/+^ (Figure 7A) and *Eln*^+/−^ (Figure 7B) P90 volume rendering shows the entire PA vascular arcade. After thresholding to remove distal vasculature, the resulting rendering (*Eln*^+/+^ Figure 7C and *Eln*^+/−^ Figure 7D) was used for analysis. Previous investigation showed that alterations in branching patterns off the MPA trunk can affect hemodynamic performance altering both flow/pressure distribution and creating areas of high wall shear stress (24, 25). The angle formed between the RPA and LPA was more acute in *Eln*^+/−^ mice compared *to Eln*^+/+^ (two-way ANOVA, genotype effect, *p* < 0.0001; Figure 7E), but no age or interactive effect was present. Multiple-comparisons revealed a reduced *Eln*^+/−^ branching angle at both P7 (*p* < 0.05) and P90 (*p* < 0.01), with a borderline difference noted at P30 (*p* = 0.08).

We know that, in addition to diminished lumen size, conduit length also increases resistance (26). Proximal pulmonary lobar arteries (see methods and Figures 7C,D for measurement boundaries) were all longer in *Eln*^+/−^ mice as compared to *Eln*^+/+^ (two-way ANOVA, genotype effect, LPLA: *p* < 0.0001, Figure 7F and RPLA: *p* < 0.001, Supplementary Figure 3A). All vessels lengthened with age (LPLA *p* < 0.0001 and RPLA *p* < 0.0001). However, *Eln*^+/−^ proximal lobar artery segments appear to lengthen more over time compared to their *Eln*^+/+^ counterparts [genotype X time interactive effect, LPLA (*p* < 0.05) and RPLA (*p* < 0.05)], suggesting another possible contributor to vascular resistance in *Eln*^+/−^ mice. Multiple-comparison testing showed longer *Eln*^+/−^ proximal arteries at P7 (*p* < 0.01), P30 (*p* < 0.001), and P90 (*p* < 0.0001) for the LPLA. Similarly, the RPLA showed an effect at P30 (*p* < 0.05) and P90 (*p* < 0.001).

Previous studies have shown increased tortuosity in systemic vessels affected by elastin insufficiency (27). The *Eln*^+/−^ pulmonary arteries exhibit evidence of mild tortuosity. The LPLA was more tortuous in *Eln*^+/−^ mice as compared to *Eln*^+/+^ (two-way ANOVA, genotype effect, *p* < 0.0001, Figure 7G). Additionally, the LPLA exhibits more tortuosity at younger ages (two-way ANOVA, age effect, *p* < 0.0001). Multiple-comparison testing showed strong effects of *Eln* genotype at P1 (*p* < 0.01) and P7 (*p* < 0.001). Although the *Eln*^+/−^ RPLA (Supplementary Figure 3B) trended toward increased tortuosity, it was not significant.

### *Eln^+/−^* Mice Exhibit Early Delays in Peripheral PA Branching, With Later Recovery and Evidence of Increased Branches

**Figure 8:**
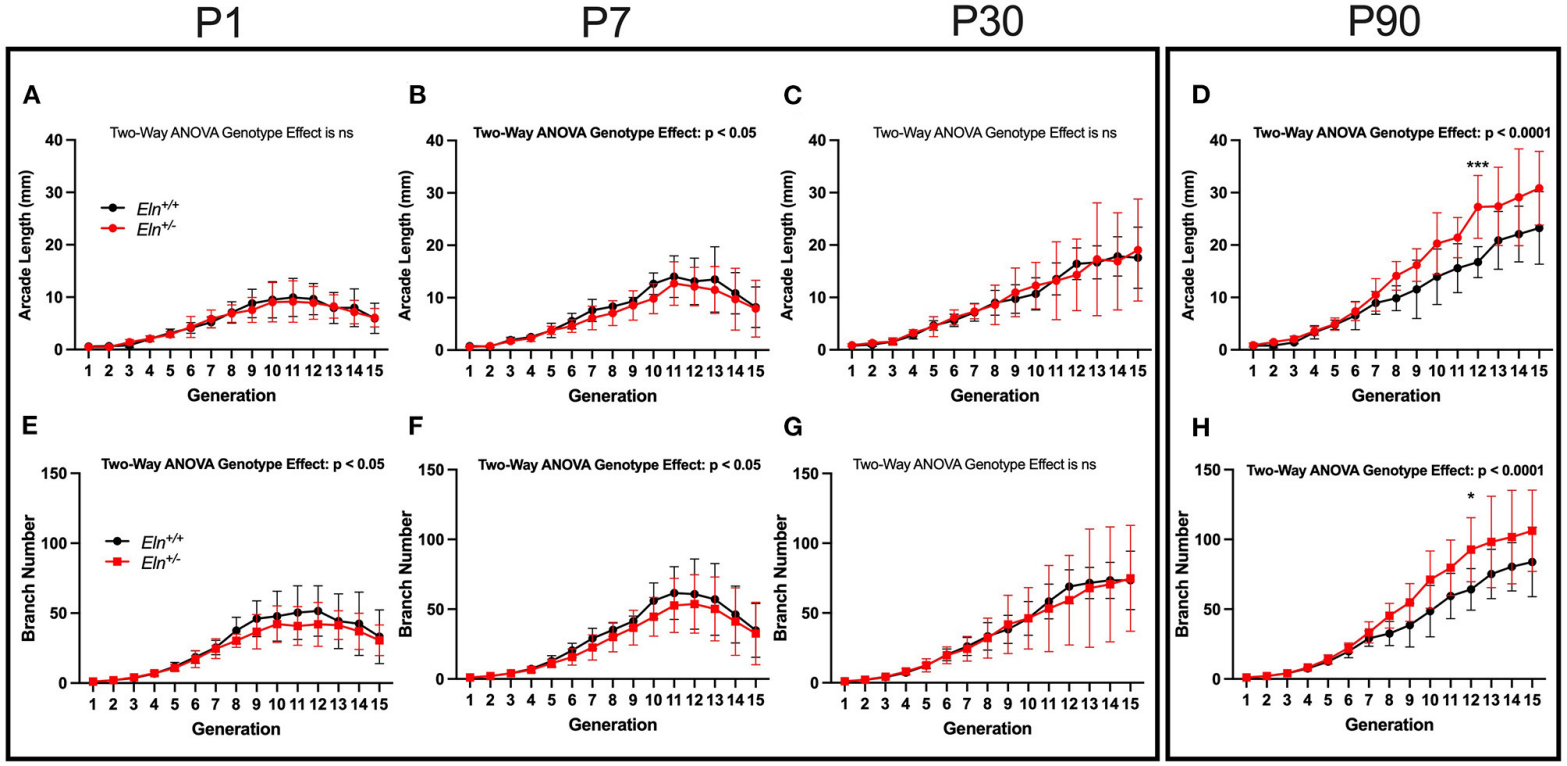
Elastin insufficiency shows diminished vascular branching early in development giving rise to increased branching in adults. Generation-specific arcade length [GSAL; **(A–D)**] and generation-specific branch number [GSBN; **(E–H)**] were evaluated at P1, P7, P30, and P90. *Eln*^+/+^ data are presented in black and *Eln*^+/−^ are in red. Mean +/- SD are shown for each age/genotype combination. Multiple comparison testing (after ANOVA) by Sidak. * *p* < 0.05, *** *p* < 0.001, and ns is not significant (*n* = 5–8/group).

For the peripheral analysis, we performed a higher resolution sub-reconstruction on the P1, P7, P30, and P90 LULFA (Figures 7C,D white arrow) and analyzed both generation specific arcade length (Figures 8A–D) and branch number (Figures 8E–H) for 15 generations. The pulmonary vascular bed can be seen as a tree that starts as a trunk (here we initiate measures in the LULFA) that then splits with successive generations. The generation-specific arcade length (GSAL) is the sum of the lengths of all the arterial segments in a given generation, while generation-specific branch number (GSBN) is the total number of branches in that generation in the vascular network supplied by the LULFA.

At P1, *Eln* genotype did not influence GSAL (two-way ANOVA repeated measures, genotype effect, *p* = NS; Figure 8A). However, P1 GSAL did differ with generation number, as it does at all ages studied (generation effect, *p* < 0.0001 in all comparisons). At P7, *Eln*^+/−^ GSAL is generally shorter than that of *Eln*^+/+^mice (genotype effect, *p* < 0.05, Figure 8B), although multiple testing shows no significant pairwise comparisons. By P30, there is again no difference in GSAL based on *Eln* genotype (genotype effect, *p* = NS, Figure 8C). However, by P90, the *Eln*^+/−^ GSAL is markedly longer than the *Eln*^+/+^ measure (genotype effect, *p* < 0.0001, Figure 8D) with an increased difference between the genotypes notable at more distal generation levels (genotype by generation interactive effect, *p* < 0.05). Pairwise comparisons are shown in the figure.

The increased GSAL could simply represent longer vascular segments (as noted in the proximal vasculature) or more branches per generation. As expected, GSBN varies by generation (two-way ANOVA repeated measures, generation effect, *p* < 0.0001 for all ages; Figures 8E–H). Decreased GSBN was noted in *Eln*^+/−^ at P1 (genotype effect, *p* < 0.05; Figure 8E) and P7 (genotype effect, *p* < 0.05; Figure 8F). As with GSAL, there was no impact of *Eln* genotype on GSBN at P30 (genotype effect, *p* = NS; Figure 8G), but a robust increase in branches noted by P90 (genotype effect, *p* < 0.0001; Figure 8H). Pairwise comparisons are shown in the figure. When the P90 GSAL is normalized to GSBL, we see that proximal segment lengths are longer in *Eln*^+/−^ pulmonary trees. In the periphery where arteriolar mechanical function is less dependent on elastin, the segment lengths per branch are similar across genotypes (See Supplementary Figure 4).

### Later Administration of Minoxidil Cannot Reverse Proximal Lobar Vessel Length, Tortuosity, LPA-RPA Angle or Generation Specific Peripheral Vascular Measures

**Figure 9:**
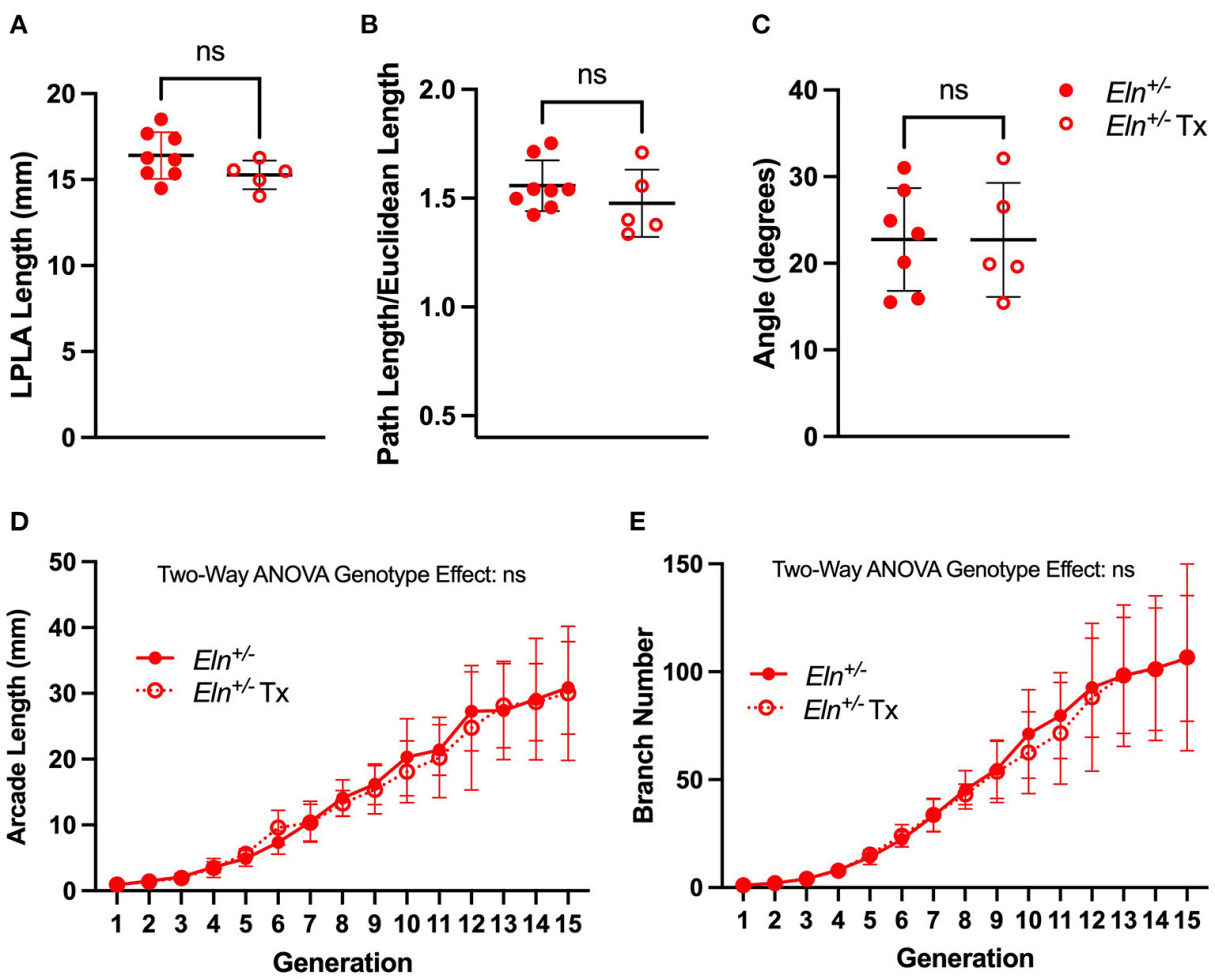
Minoxidil does not impact post-natal branching patterns in *Eln*^+/−^ mice. A lack of treatment effect for minoxidil is evident in P90 left pulmonary lobar artery (LPLA) length **(A)**, tortuosity **(B)** and LPA-RPA branching angle **(C)**. Similarly, no treatment differences are noted in generation-specific arcade length **(D)** or branch number **(E)**. Untreated *Eln*^+/−^ values are in closed circles and treated *Eln*^+/−^ values are depicted with open circles. In **(A–C)**, a mean line +/- SD are shown for each timepoint/genotype combination. In **(D,E)**, mean +/- SD are shown for all points. ns is not significant and *n* = 5–6/group for **(D,E)**.

As in humans with WBS and elastin insufficiency, typically diagnosed postnatally after the cardiovascular changes become apparent, we initiated Tx in mice post-weaning (~P21–P90). As such, we only evaluated the Tx effect at P90 using polymer injected lung mounts imaged by μCT. In this case, Tx did not reduce the length of any *Eln*^+/−^ lobar arteries (unpaired *t*-test, *p* = NS; LPLA Figure 9A and RPLA Supplementary Figure 5A). Additionally, treatment had no impact on vessel tortuosity (unpaired *t-*test, p=NS; LPLA Figure 9B and RPLA Supplementary Figure 5B).

Similarly, post-weaning Tx did not widen the acute *Eln*^+/−^ LPA-RPA angle (unpaired *t*-test, Figure 9C). Replication of LPA-RPA angle measurement using *in vivo* CT angiogram revealed similar findings across all genotypes (One way ANOVA, followed by pairwise comparisons, Supplementary Figure 5C), suggesting the findings are not just a byproduct of our casting process.

Finally, evaluation of the peripheral arcade in polymer injected lungs by two-way ANOVA repeated measures showed the previous patterns of generation-level GSAL and GSBN (Figures 9D,E, *p* < 0.0001 in both evaluations). However, there was no Tx effect.

## Discussion

Elastin haploinsufficiency causes significant morbidity and mortality in affected individuals. While SVAS is the most common vascular lesion in people with WBS (OMIM #194050) and *ELN*-related SVAS (OMIM #185500), ~40–60% of individuals with these conditions have stenosis in the pulmonary tree (3, 4). The natural history for pulmonary vascular disease in elastin insufficiency is for eventual resolution in most mild cases, while moderate or severe disease usually requires surgical intervention (6). Smooth muscle cells in elastin insufficient vessels display abnormal circumferential growth (28) and fail to remodel appropriately with attempted percutaneous dilation (4, 29). Success rates for primary catheter-based palliation of branch PA disease have been reported at 51%, with frequent need for subsequent surgical intervention (30, 31). As such these procedures are now predominantly used as a temporizer before or between surgeries. Because the condition tends to impact multiple levels of the pulmonary vasculature from the hilum into the periphery, the surgery is exceedingly complex and the number of centers willing to attempt such approaches is vanishingly small. The reports of successful palliation in these centers, however, are encouraging (7, 32). Still, there is room for alternate approaches, if only because the availability of such specialized care is so limited. Additionally, data presented here show persistently elevated RVSPe in a subset of patients even after intervention. Figure 1, for example, includes one individual who benefited from extensive PA reconstruction beyond the hilum, but whose pressures remain high even though the proximal vascular measurements are relatively normal. Consequently, additional therapeutic options should be explored. The aim of this study was to characterize *Eln*^+/−^ pulmonary vasculature structure from the branch pulmonary arteries to the periphery and to evaluate whether treatment with K_ATP_ channel opener, minoxidil, could mitigate pulmonary vascular abnormalities seen in *Eln*^+/−^ mice. Minoxidil was initially developed as an anti-hypertensive owing to its inhibition of excitation-contraction coupling in muscle resulting in primary vasodilatory effect. However, subsequent studies showed that when given chronically, it induced expression of connective tissue genes that stabilize the vessel in its new dilated state.

Our analysis shows that *Eln*^+/−^ mice display cardiovascular defects at all levels of the pulmonary vascular tree. RVSP is increased in the *Eln*^+/−^, as assessed by both direct invasive catheterization (Figure 2A) and indirect echocardiographic assessment (Figure 6E). Furthermore, the right heart exhibits increased normalized mass (Figure 2C). Of note our data exhibit a smaller elastin insufficiency-associated increase in RVSP (9.94 (male) and 7.07 (female), and 8.62 (male and female combined) mmHg in this study, respectively vs. 34.8 mm Hg in Shifren et al. (33)). Reasons for this difference in magnitude may include genetic background, anesthetic choice, catheter characteristics, and general drift in mutant pressure over time. The initial publication of the *Eln*^+/−^ model in 1998 showed an average systemic systolic blood pressure of 165 mmHg (34), while more recent publications report an average of 127 mmHg (8), potentially implicating epigenetic phenomena (35).

*In vivo* CT angiogram and *ex vivo* pressure-diameter testing by pressure myography confirm reduced *Eln*^+/−^ inner and outer PA diameters, with the decreased slope of the myography-assessed pressure-diameter curve over the working range of the vessel (0–40 mm Hg in the *Eln*^+/−^ mouse, Figure 3A) suggesting increased arterial stiffness. Likewise, histological analysis revealed medial hypertrophy in *Eln*^+/−^ PAs (Figure 3H). Wall thickening such as this is commonly seen in humans with pulmonary vascular disease (36, 37) and may synergize with the primary elastin insufficiency defect to increase stiffness. Such features may suggest that the *Eln*^+/−^ vasculopathy may limit vascular carrying capacity (both in the form of blood volume and oxygen/nutrient delivery). Indeed, previous studies evaluating the brain perfusion in the *Eln*^+/−^ mouse also endorse reduced end organ flow (8).

**Figure 10:**
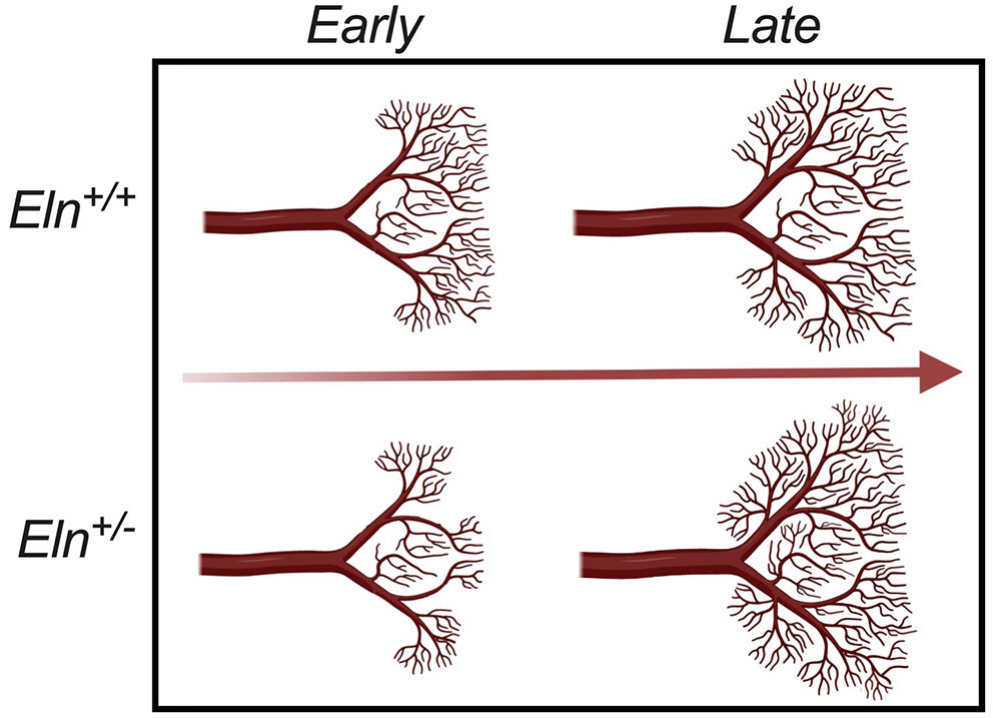
Developmental time-course of Elastin haploinsufficient vasculature. Early (P1 and P7) in development, distal *Eln*^+/−^ PAs exhibit less branching than the *Eln*^+/+^. As the lungs grow and mature, the distal vasculature in the *Eln*^+/−^ branch more - catching and eventually surpassing the *Eln*^+/+^ counterpart later (P30 and P90) in development. This suggests a possible compensatory response to elastin insufficiency mediated changes in pulmonary physiology that occur over a wide developmental window.

In addition to the proximal caliber measures, our latex injection-μCT studies yielded evidence of abnormal proximal branching angles (Figure 7E), increased pulmonary arterial segment length (Figure 7F), increased tortuosity (Figure 7G) and evidence of altered peripheral branching (Figure 8). Figure 10 illustrates the two notable developmental phases. Early in post-natal life (P1 and P7), the *Eln*^+/−^ LULFA displays reduced branching. But then later, as the lungs grow and mature, the rate of branching increases, catching (P30) and eventually surpassing (P90) their *Eln*^+/+^ counterpart. Reduced early branching was previously reported by Hilgendorff et al., who assessed pulmonary microvasculature in ventilated and unventilated P5-7 *Eln*^+/−^ mice, showing an overall reduction in small vessel (<20μm) number, relative to their *Eln*^+/+^ counterparts (38), with no change in alveolar number. The authors suggest that decreased arteriolar number may contribute to early pulmonary hypertension in their model. Indeed, many of the measures shown here may contribute to increased pulmonary pressure and possibly increased pulmonary vascular resistance in the *Eln*^+/−^ pup. While we were unable to measure mouse pulmonary vascular resistance (PVR) directly, we did note several indirect measures suggestive of elevated PVR in mutant mice, including systemic notching and reduced PAAT, even at later ages after the branch number had normalized or surpassed the *Eln*^+/+^ (39–41).

The phenomenon of altered branching remains unexplained. It is possible that early reduced vessel number represents a primary developmental abnormality of the *Eln*^+/−^, with later vessel proliferation a compensatory response to decreased tissue perfusion. It is also possible that the later change in vascular patterning instead indicates a response to elastin mediated disease in the lung parenchyma. Recent studies have begun to show evidence of air trapping and obstruction in humans with WBS and elastin insufficiency (42, 43) and altered lung compliance in the *Eln*^+/−^ mouse (44, 45). More investigation is needed to understand this complex phenotype and how it may relate to the resolution of mildly elevated pulmonary artery pressures seen in many children with WBS over their first few years of life.

In this study, we also evaluated sex as a biological variable. In general, genotype X sex interactions were rare. The most notable exception was in medial thickness. While thickened abdominal aortic media has been reported in Wagenseil et al. (46), not all authors have reported thicker *Eln*^+/−^ vessels, especially when using full wall, rather than media-only measures (33, 47, 48). Interestingly, our data showed more robust medial thickening in male *Eln*^+/−^ pulmonary arteries than female, although both sexes exhibited this phenotype (Figure 3H). This sex effect is clinically relevant as males with elastin insufficiency as a result of WBS are reported to have more severe vascular disease than females (11). A recent genetic study confirmed this sex difference in SVAS outcomes (49), but it has not been specifically examined in terms of pulmonary vascular outcomes. Systemic arterial wall thickness, in general, has been reported as higher in adult and pediatric males, and increases with age (50). As noted above, medial thickening is often seen in PAs that have been chronically exposed to higher pulmonary pressures. Indeed, male *Eln*^+/−^ do exhibit the highest RVSP of all groups. It is possible that there are certain pressure thresholds reached by male *Eln*^+/−^, but not female *Eln*^+/−^ mice that accelerate this outcome. Additional investigation of molecular signatures that differ between *Eln*^+/−^ male and female pulmonary arteries is warranted as it may reveal factors that could serve as rational therapeutics for this condition.

Finally, and most importantly, we endeavored to evaluate minoxidil, a K_ATP_ channel opener and vasodilator (51, 52), as a treatment of pulmonary vasculopathy in people with WBS. Previous work with this drug showed that chronic administration increased arterial caliber in *Eln*^+/−^ mice, decreased systemic blood pressure and led to systemic large artery remodeling, first by vasodilation and then with increased elastin and other connective tissue molecule deposition in the extracellular space (8). Moreover, these changes in vessel phenotype and composition persisted weeks after the drug had been removed. Similar functional improvements were seen in the treatment of wildtype mice who had experienced elastin loss due to age (9). A clinical study using minoxidil in a small number of individuals with WBS showed no improvement of the intima-media thickness in the carotid artery. The same study did, however show an increase in carotid lumen diameter in the same participants (10), suggesting that it may still prove useful in the treatment of elastin-mediated disease if used for different indications.

As such, we compared pulmonary vascular findings in *Eln*^+/−^ mice administered minoxidil in their drinking water from weaning until 3 months of age to an untreated cohort. Encouragingly, we saw a drop in RVSP (Figure 5A) and an increase in proximal PA caliber by *in vivo* CT and *ex vivo* myography and histology (Figure 4) with minoxidil. However, markers suggestive of increased pulmonary vascular resistance in *Eln*^+/−^ mice [PAAT and systolic notching, (41)] were not impacted by the drug (Figures 6D–E). The lack of improvement in these areas, underscores the multi-dimensional impact of elastin insufficiency on vessel physiology. Branching angle, arterial segment length, tortuosity, and differences in peripheral branching can all influence flow and vascular resistance patterns (24–26) and had already been established as abnormal in the *Eln*^+/−^ as early as P7 (Figures 7E–G and Figure 8). While slight trends toward improvement of these measures were noted with Tx initiated at weaning (Figures 9A–C), the effect was not statistically significant, potentially because therapy was initiated too late. Earlier intervention should be prioritized in future trials.

In addition to the noted vascular features, minoxidil also increased heart mass. Given that minoxidil successfully decreased RVSP and increased lumen size, one would have predicted decreased RV mass to result from this. Interestingly, multiple authors do note cardiac hypertrophy as a common off-target effect of this drug, specifically in rodent models (53, 54). Review of those studies shows a trend toward larger cardiomyocyte cross-sectional area in treated WT animals, with more severe features noted in the RV. Subsequent work using K_ATP_ gain of function mutants suggest increased L-type calcium current accompanies increased K_ATP_ current, suggesting a possible mechanistic impetus underlying cardiac hypertrophy seen here (55–57). Regardless, we believe this to be an off-target effect of the medication that appears most pronounced in rodents. Of note, cardiac hypertrophy was not noted in the previous minoxidil trial in WBS (10), nor was it seen consistently in human subjects with Cantu Syndrome, which arises from K_ATP_ gain of function channel mutations (57).

Taken together, these findings suggest that elastin insufficiency produces a complex collection of vascular findings in the lung that are not limited to the proximal vasculature. Thus, optimal treatment for vascular disease in elastin insufficiency will require interventions which impact vessels at multiple levels and coincide with relevant developmental timelines. Our data show that minoxidil effectively improves proximal arterial caliber in elastin haploinsufficiency, but when delivered post-weaning in mice does not completely reverse vascular abnormalities. Consequently, to be used therapeutically, early diagnosis and treatment of elastin mediated pulmonary disease may yield more efficacious results. Our experience with treatment of mice in systemic circulation suggested that minoxidil could induce transcriptional changes that led to meaningful remodeling of *Eln*^+/−^ vessels. As such, minoxidil could be considered for the treatment of younger children with severe pulmonary vascular disease as an adjunct to surgical intervention to improve lumen dimensions beyond the surgical field. Earlier treatment may influence other still developing contributors to vascular resistance such as branching angle and segment length. While it is likely that the hypertrophy seen here is a consequence of peculiarities of mouse physiology, cardiac size should be monitored and long-term use minimized to avoid off-target drug effects including hirsutism and skeletal bone remodeling.

## Data Availability Statement

The raw data supporting the conclusions of this article will be made available by the authors, without undue reservation.

## Ethics Statement

The studies involving human participants were reviewed and approved by Institutional Review Board of the National Institutes of Health. Written informed consent to participate in this study was provided by the participants' legal guardian/next of kin. The animal study was reviewed and approved by Institutional Animal Care and Use Committee of the National Heart Lung and Blood Institute or Washington University School of Medicine.

## Author Contributions

BK, RK, and LG: conceptual/experimental design. RK, LG, EK, MK, DD, DS, ZY, MC, Y-PF, FC, M-LN, SO, JF, and NR: data collection. RK, LG, EK, MK, DD, DS, MC, Y-PF, FC, M-LN, ML, and BK: data analysis. RK, BK, and LG: wrote the manuscript. RK, BK, LG, EK, MK, DD, DS, ZY, MC, Y-PF, FC, M-LN, SO, JF, NR, and ML: edited the manuscript. All authors contributed to the article and approved the submitted version.

## Funding

Funding for this work was provided by the Division of Intramural Research of the National Heart, Lung, and Blood Institute through grants ZIA HL006210 and ZIA HL-006247.

## Conflict of Interest

The authors declare that the research was conducted in the absence of any commercial or financial relationships that could be construed as a potential conflict of interest.

## Publisher's Note

All claims expressed in this article are solely those of the authors and do not necessarily represent those of their affiliated organizations, or those of the publisher, the editors and the reviewers. Any product that may be evaluated in this article, or claim that may be made by its manufacturer, is not guaranteed or endorsed by the publisher.

## Glossary

WBS: Williams Beuren syndrome
CT: Computed Tomography
μCT: micro-CT
PA: Pulmonary Artery
RVSPe: Right Ventricular Pressure estimate
RVSP: Right Ventricular Systolic Pressure
SVAS: Supravalvar Aortic Stenosis
TR: Tricuspid Regurgitation
LPA: Left Pulmonary Arteries
MPA: Main Pulmonary trunk Arteries
BM: Body Mass
LV: Left Ventricle/septum
TL: Tibia Length
RVHM: Right Ventricular Heart Mass
LVHM: Left Ventricular Heart Mass
RPA: Right Pulmonary Artery
RVOT: Right Ventricular Outflow Tract
PAAT: Pulmonary Artery Acceleration Time
PW: Pulsed Wave
P: Postnatal
LULFA: Left Upper Lobe Feed Artery
LPLA: Left Pulmonary Lobar Artery
RPLA: Right Pulmonary Lobar Artery
Tx: Treatment
LVESV: Left Ventricular End Systolic Volume
LVEDV: Left Ventricular End Diastolic Volume
LVIDs: Left Ventricular Inner Diameter Systole
LVIDs: Left Ventricular Inner Diameter Diastole
LVAWs: Left Ventricular Anterior Wall Systole
LVAWd: Left Ventricular AnteriorWall Diastole
LVPWs: Left Ventricular Posterior Wall Systole
LVPWd: Left Ventricular Anterior Wall Diastole
IVSs: Intraventricular Septum Systole
IVSd: Intraventricular Septum Diastole
LVM: calculated Left Ventricular Mass
LVMI: calculated Left Ventricular Mass Indexed to body mass
LVEF: Left Ventricular Ejection Fraction
LVFS: Left Ventricular Fractional Shortening
LVOT: Left Ventricular Outflow Tract
RVDP: Right Ventricular Diastolic Pressure
HR: Heart rate
GSAL: Generation-Specific Arcade Length
GSBN: Generation-Specific Branch Number
COPD: Chronic Obstructive Pulmonary Disease
SD: Standard Deviation.
