# Supplementary material for: Elastin Insufficiency Confers Proximal and Distal Pulmonary Vasculopathy in Mice, Partially Remedied by the KATP Channel Opener Minoxidil: Considerations and Cautions for the Treatment of People With Williams-Beuren Syndrome
Source: Front Cardiovasc Med. 2022 May 19;9:886813. doi: 10.3389/fcvm.2022.886813 (PMC9160528; doi:10.3389/fcvm.2022.886813)
Supplement: Supplementary file 1 [file Data_Sheet_1.pdf]

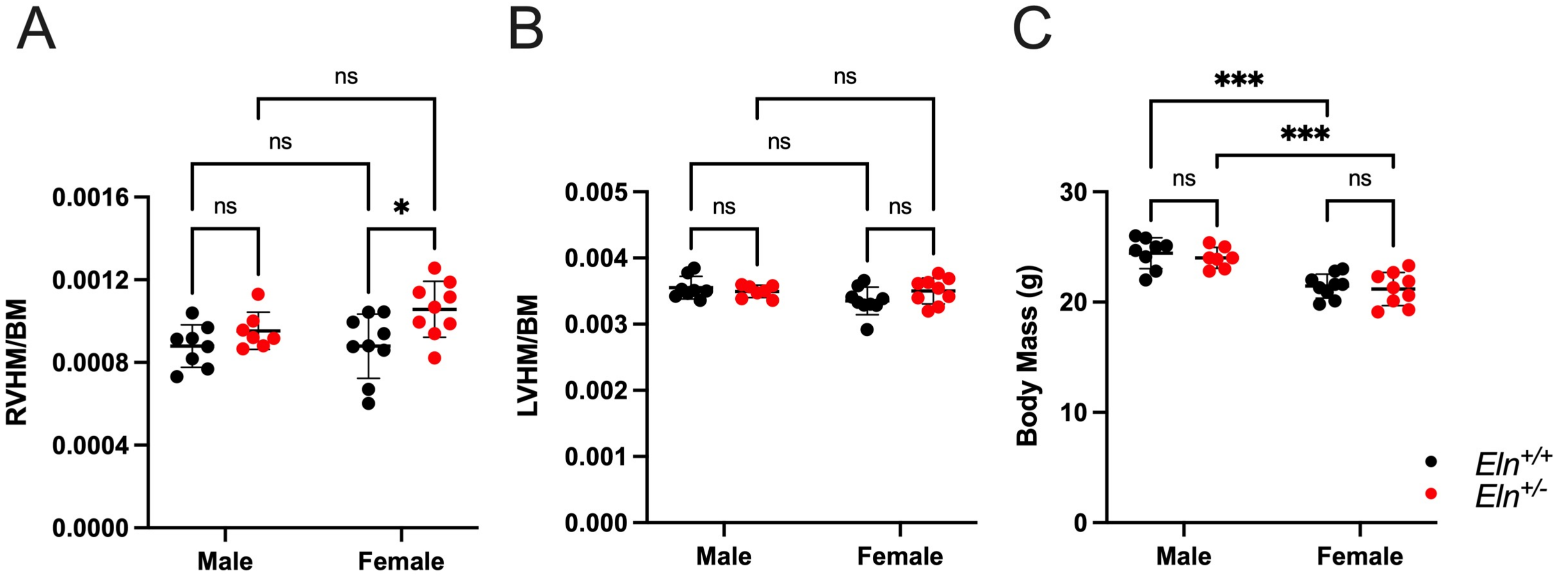

Supplemental Figure 1. Sex and Eln genotype differences in hemodynamic parameters. Panel A-C report heart mass as normalized to body mass findings. A: Right ventricular heart mass/body mass (RVHM/BM), B: Left ventricular heart mass/body mass (LVSHM/BM, this measure includes the intraventricular septum and the LV in the LVSHM measure), C: Body Mass. *Eln*$^{+/+}$ data are presented in black and *Eln*$^{+/-}$ in red in all figures. A mean line +/- SD is shown for each sex/genotype combination. Multiple comparison testing (after ANOVA) by Tukey. * $p<0.05$, *** $p<0.001$, and ns is not significant.

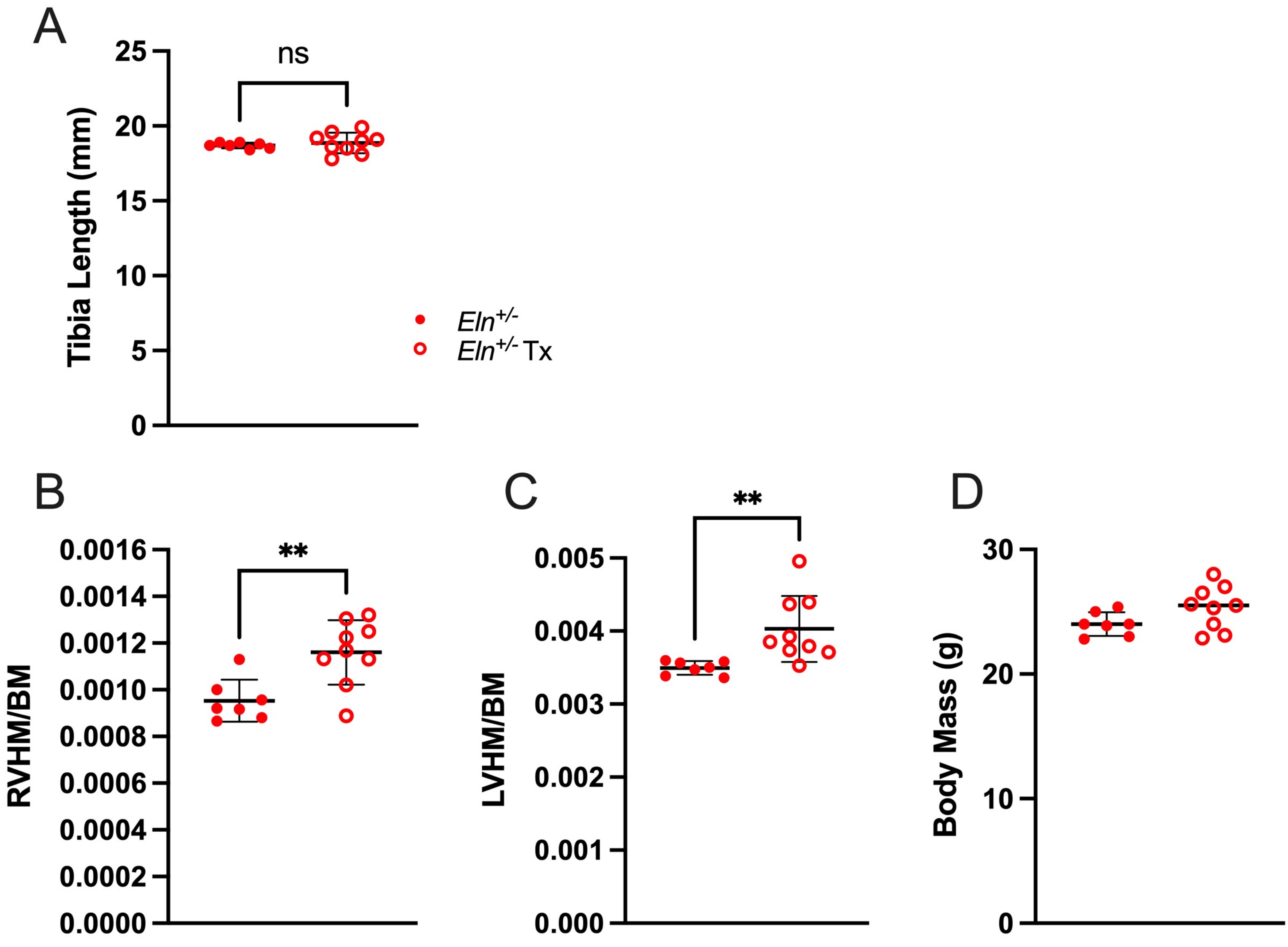

Supplemental Figure 2: Minoxidil increases heart size in *Eln*$^{+/-}$ mice. Panel A shows tibia length. Panel B-D report heart mass as normalized to body weight findings. B: Right ventricular heart mass/body mass (RVHM/BM,) C: Left ventricular heart mass/body mass (LVHM/BM), D: body mass. *Eln*$^{+/-}$ data are presented as red closed circles and *Eln*$^{+/-}$ treated (Tx) as open circles. A mean line +/- SD is shown at the median for each treatment/genotype combination. Testing by Mann-Whitney. ** p<0.01 and ns is not significant.

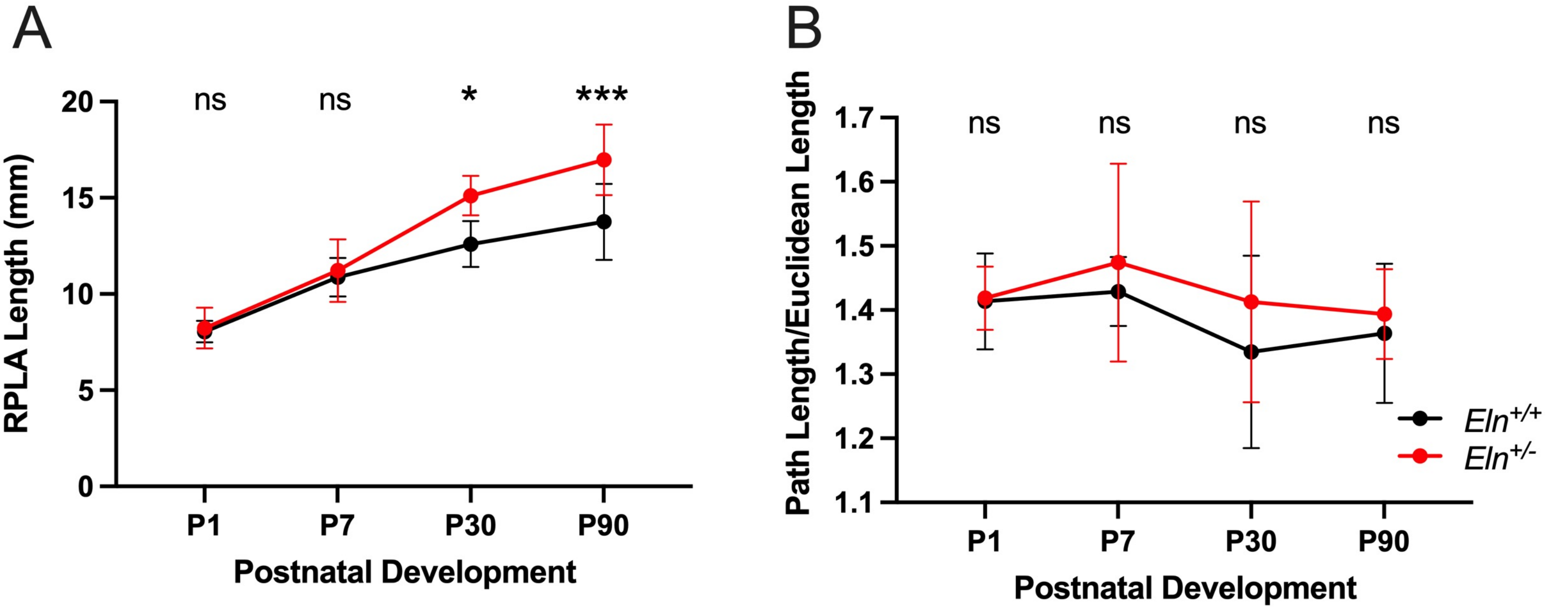

Supplemental Figure 3: Elastin insufficiency increases right pulmonary lobar artery length and shows signs of increased tortuosity. Similar to the LPLA, the RPLA length (A) and tortuosity (B) are reported at P1, P7, P30 and P90. *Eln*$^{+/+}$ data are presented in black and *Eln*$^{+/-}$ are in red. Mean +/- SD are shown for each age/genotype combination. Multiple comparison testing (after ANOVA) by Sidak. * $p<0.05$, *** $p<0.001$, and ns is not significant (n = 5-8/group).

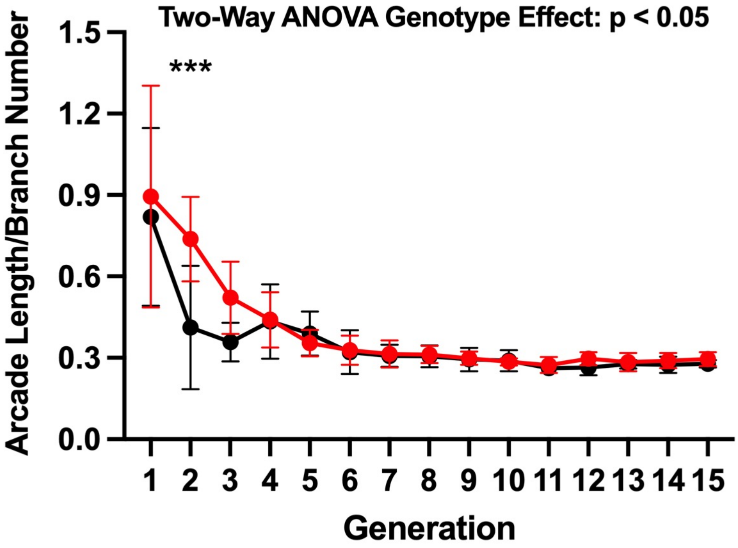

Supplemental Figure 4: Proximal arterial segments in *Eln*$^{+/-}$ mice are longer while more distal vessels show no apparent differences in length. When generation-specific arcade length (GSAL) is normalized to generation-specific branch number (GSBN), the *Eln*$^{+/-}$ proximal segments are longer than the *Eln*$^{+/+}$. No apparent difference is evident in the distal vasculature. *Eln*$^{+/+}$ are depicted using closed black circles and *Eln*$^{+/-}$ values are closed red circles. Mean +/- SD are shown for each timepoint/genotype. Multiple comparison testing (after ANOVA) by Sidak. *** $p < 0.001$.

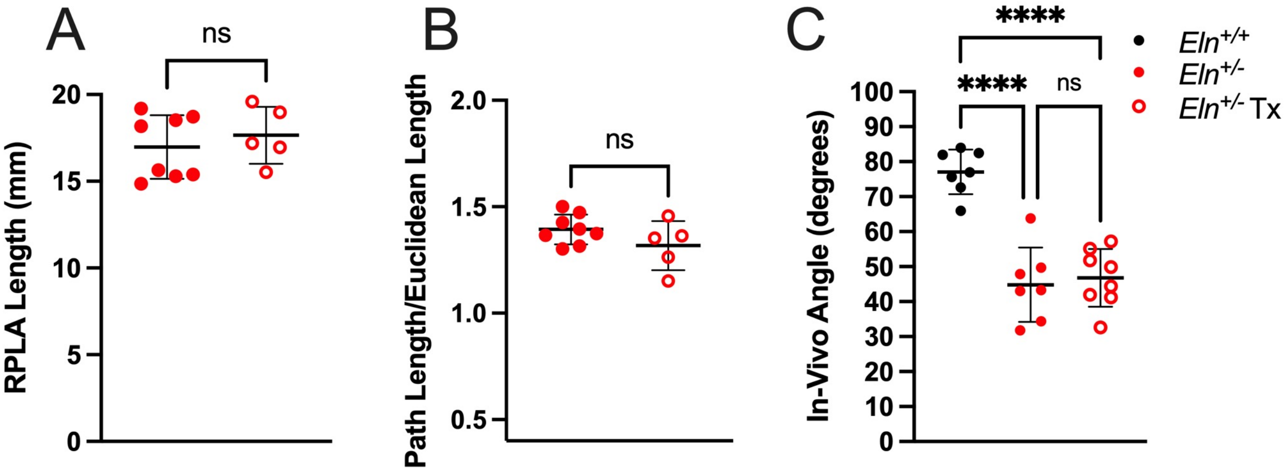

Supplemental Figure 5: Minoxidil does not impact post-natal branching patterns in *Eln*$^{+/-}$ mice. Similar to the LPLA, a lack of treatment effect for minoxidil is apparent in both RPLA length (A) and tortuosity (B). Utilizing *in vivo* uCT, Panel C confirms the *Eln*$^{+/-}$ LPA-RPA branching angle is significantly more acute that the *Eln*$^{+/+}$. However, minoxidil treatment does not alter the *Eln*$^{+/-}$ branching angle. *Eln*$^{+/+}$ are depicted using closed black circles, untreated *Eln*$^{+/-}$ values are closed red circles, and treated *Eln*$^{+/-}$ values are depicted with open red circles. Mean lines +/- SD are shown for each timepoint/genotype combination. Unpaired t-tests were performed for panels A and B. Multiple comparison testing (after ANOVA; C) by Tukey **** $p < 0.0001$ and ns is not significant.

Supplemental Table 1: Demographic Features for Human Studies Participants. Data are shown for individuals with WBS or controls who participated in echocardiogram or CT angiogram studies. The CT participants are a subset of the echocardiogram group. ND: not determined.

Echocardiogram	WBS	Control	P value
Number of participants with TRV_{max} data	20	11	ND
Age (y) mean +/-SD (range)	9.3 +/- 3.5 y (3.4-17.8)	12.0 +/-4.2 y (4.4-16.8)	0.29
Number (%) Female	9 (45%)	4 (37%)	0.72
Body Mass Index mean +/- SD	16.9 +/- 3.0	18.9 +/- 3.3	0.26
Number (%) with procedures on PA(s) only	2 (10%)	0 (0%)	ND
Number (%) with procedures on aorta only	2 (10%)	0 (0%)	ND
Number (%) with procedures on both aorta and PA(s)	5 (25%)	0 (0%)	ND
Number (%) with any of the above procedures	9 (34.3%)	0 (0%)	ND
Number with TRV_{max} of original echo set (%)	20/35 (57.1%)	11/13 (84.6%)	0.10

CT angiogram	WBS
Number of TRV_{max} positive participants with unsedated CT data	11
Age (y) mean +/-SD (range)	10.5 +/- 3.4 (6.8-17.7)
Number (%) Female	6 (54%)
Body Mass Index mean +/- SD	17.6 +/- 3.7
Number (%) with procedures on PA(s) only	2 (18%)
Number (%) with procedures on aorta only	1 (9%)
Number (%) with procedures on both aorta and PA(s)	4 (36%)
Number (%) with any of the above procedures	9 (64%)

Echo Parameter	Eln+/+	Eln+/-	Eln+/-Tx	Eln+/+ vs Eln+/-	Eln+/+ vs Eln+/-Tx	Eln+/- vs Eln+/-Tx
**Body Mass (g)**	24.40 +/- 1.48	23.94 +/- 1.01	24.99 +/- 1.51	p = 0.79	p = 0.68	p = 0.29
**Heart Rate (BPM)**	515.10 +/- 31.27	477.0 +/- 35.36	517.25 +/- 41.00	p = 0.14	p = 0.99	p = 0.10
**LVESV (µl)**	16.53 +/- 2.64	17.31 +/- 1.82	18.16 +/- 2.74	p = 0.81	p = 0.41	p = 0.77
**LVEDV (µl)**	49.90 +/- 8.61	55.39 +/- 4.77	60.09 +/- 6.80	p = 0.29	**p = 0.02**	p = 0.37
**LVIDs (mm)**	2.21 +/- 0.14	2.26 +/- 0.09	2.3 +/- 0.15	p = 0.80	p = 0.41	p = 0.78
**LVIDd (mm)**	3.46 +/- 0.25	3.62 +/- 0.13	3.75 +/- 0.18	p = 0.25	**p = 0.02**	p = 0.41
**LVAWs (mm)**	1.19 +/- 0.02	1.20 +/- 0.02	1.37 +/- 0.07	p = 0.96	**p < 0.0001**	**p < 0.0001**
**LVAWd (mm)**	0.78 +/- 0.02	0.78 +/- 0.04	0.91 +/- 0.06	p = 0.99	**p < 0.0001**	**p < 0.0001**
**LVPWs (mm)**	1.17 +/- 0.02	1.19 +/- 0.03	1.34 +/- 0.07	p = 0.76	**p < 0.0001**	**p < 0.0001**
**LVPWd (mm)**	0.78 +/- 0.02	0.79 +/- 0.04	0.91 +/- 0.06	p = 0.92	**p < 0.0001**	**p = 0.0002**
**IVSs (mm)**	1.19 +/-0.02	1.20 +/- 0.02	1.37 +/- 0.07	p = 0.95	**p < 0.0001**	**p < 0.0001**
**IVSd (mm)**	0.79 +/- 0.02	0.78 +/- 0.04	0.90 +/- 0.06	p = 0.98	**p = 0.0002**	**p = 0.0002**
**LVM (mg)**	79.96 +/- 4.77	80.05 +/- 5.19	97.70 +/- 10.17	p = 0.99	**p = 0.0004**	**p = 0.0003**
**LVMI (mg/g)**	3.29 +/- 0.33	3.35 +/- 0.24	3.93 +/- 0.54	p = 0.96	**p = 0.01**	**p = 0.02**
**LVEF (%)**	66.70 +/- 1.33	68.75 +/- 1.31	69.68 +/- 3.35	p = 0.21	**p = 0.04**	p = 0.69
**LVFS (%)**	36.04 +/- 1.15	37.75 +/- 0.98	38.60 +/- 2.65	p = 0.18	**p = 0.03**	p = 0.61
**LVOT Peak Gradient (mmHg)**	5.90 +/- 0.57	5.10 +/- 0.78	6.54 +/- 1.08	p = 0.21	p = 0.38	**p = 0.01**
**LVOT Peak Velocity (mm/s)**	1211.67 +/- 62.17	1124.71 +/- 88.99	1277.86 +/- 104.53	p = 0.19	p = 0.39	**p = 0.01**
**RVOT Peak Gradient (mmHg)**	2.19 +/- 0.09	2.35 +/- 0.20	2.16 +/- 0.38	p = 0.45	p = 0.98	p = 0.33
**RVOT Peak Velocity (mm/s)**	740.57 +/- 11.47	761.88 +/- 33.77	733.88 +/- 62.09	p = 0.60	p = 0.95	p = 0.40
**PAAT (ms)**	16.74 +/- 0.40	13.80 +/- 1.20	13.98 +/- 1.39	**p = 0.0002**	**p = 0.0003**	p = 0.94

Supplemental Table 2. Echocardiographic summary of elastin haploinsufficent mice and the relative effects of treatment with Minoxidil. LVESV left ventricular end systolic volume, LVEDV left ventricular end diastolic volume, LVIDs left ventricular inner diameter systole, LVIDd left ventricular inner diameter diastole, LVAWs left ventricular anterior wall systole, LVAWd left ventricular anterior wall diastole, LVPWs left ventricular posterior wall systole, LVPWd left ventricular anterior wall diastole, IVSs intraventricular septum systole, IVSd intraventricular septum diastole, LVM calculated left ventricular mass, LVMI calculated left ventricular mass indexed to body mass, LVEF left ventricular ejection fraction, LVFS left ventricular fractional shortening, LVOT left ventricular outflow tract, RVOT right ventricular outflow tract, PAAT pulmonary artery acceleration time. All statistics were performed using one-way ANOVA with Tukey's multiple comparisons testing. Statistically significant values are in red. Mean values +/- SD represented.
